# CSB and SMARCAL1 compete for RPA32 at stalled forks and differentially control the fate of stalled forks in BRCA2-deficient cells

**DOI:** 10.1093/nar/gkae154

**Published:** 2024-02-28

**Authors:** Nicole L Batenburg, Dana J Sowa, John R Walker, Sara N Andres, Xu-Dong Zhu

**Affiliations:** Department of Biology, McMaster University, Hamilton, Ontario L8S 4K1, Canada; Department of Biochemistry and Biomedical Sciences, McMaster University, Hamilton, Ontario L8S 4K1, Canada; Department of Biology, McMaster University, Hamilton, Ontario L8S 4K1, Canada; Department of Biochemistry and Biomedical Sciences, McMaster University, Hamilton, Ontario L8S 4K1, Canada; Department of Biology, McMaster University, Hamilton, Ontario L8S 4K1, Canada

## Abstract

CSB (Cockayne syndrome group B) and SMARCAL1 (SWI/SNF-related, matrix-associated, actin-dependent, regulator of chromatin, subfamily A-like 1) are DNA translocases that belong to the SNF2 helicase family. They both are enriched at stalled replication forks. While SMARCAL1 is recruited by RPA32 to stalled forks, little is known about whether RPA32 also regulates CSB’s association with stalled forks. Here, we report that CSB directly interacts with RPA, at least in part via a RPA32C-interacting motif within the N-terminal region of CSB. Modeling of the CSB-RPA32C interaction suggests that CSB binds the RPA32C surface previously shown to be important for binding of UNG2 and SMARCAL1. We show that this interaction is necessary for promoting fork slowing and fork degradation in BRCA2-deficient cells but dispensable for mediating restart of stalled forks. CSB competes with SMARCAL1 for RPA32 at stalled forks and acts non-redundantly with SMARCAL1 to restrain fork progression in response to mild replication stress. In contrast to CSB stimulated restart of stalled forks, SMARCAL1 inhibits restart of stalled forks in BRCA2-deficient cells, likely by suppressing BIR-mediated repair of collapsed forks. Loss of CSB leads to re-sensitization of SMARCAL1-depleted BRCA2-deficient cells to chemodrugs, underscoring a role of CSB in targeted cancer therapy.

## Graphical Abstract

**Graphical Abstract:**
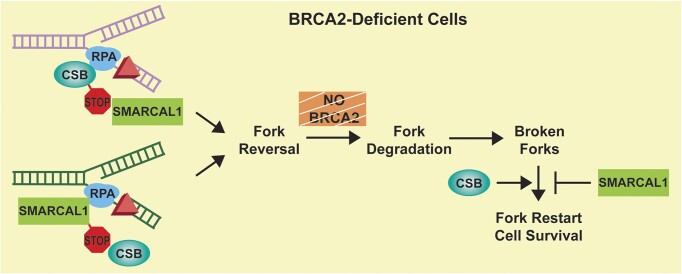

## Introduction

Progression of DNA replication forks is frequently challenged by both endogenous and exogenous sources of DNA damage, leading to replication stress, a known driver of genomic instability (1). Replication stress is frequently observed during the development of human cancers (2–4). Mammalian cells have evolved several interconnected responses to replication stress (5–7). Uncoupling of replication forks generates single-strand DNA (ssDNA), which is coated by replication protein A (RPA). RPA bound to ssDNA recruits and activates the ataxia telangiectasia and Rad3-related (ATR) checkpoint kinase (8), which in turn stabilizes stalled forks, prevents origin firing, and promotes cell cycle arrest. Stalled replication forks can be remodeled into reversed forks, a process known as fork reversal that is thought to serve as a protective mechanism, allowing resumption of DNA synthesis without chromosome breakage (9). Furthermore, several DNA damage tolerance pathways can be engaged to allow DNA replication to continue in the presence of DNA lesions, including translesion synthesis (TLS), template switching, and PRIMPOL-mediated fork repriming. Recent studies suggest that fork reversal and fork repriming are competing options in response to replication stress (10,11).

SMARCAL1 (SWI/SNF-related, matrix-associated, actin-dependent, regulator of chromatin, subfamily A-like 1) is a DNA translocase that belongs to the SNF2 helicase family (12). SMARCAL1 is associated with replication forks in unperturbed cells and this association is further enhanced upon replication stress through its binding to RPA, a heterotrimer consisting of RPA70, RPA32, and RPA14, via a RPA32 interaction motif within the N-terminus of SMARCAL1 (13–15). SMARCAL1 anneals RPA coated ssDNA (16) and catalyzes remodeling of stalled forks into reversed forks (15). SMARCAL1 restrains progression of replication forks upon replication stress (17,18), which is attributed to its role in fork reversal. In the absence of SMARCAL1, PRIMPOL-mediated fork repriming is responsible for unrestrained progression of replication forks upon replication stress (10). Fork reversal can become a source of DNA damage when fork stability is compromised under pathological conditions such as deficiency in BRCA1 and BRCA2 (19–21). In this situation, reversed forks are degraded by MRE11 and EXO1 nucleases or cleaved by MUS81 (22–24). Loss of SMARCAL1 is found to both restore fork stability and confer chemoresistance in cells lacking BRCA1 or BRCA2 (19,25) although separate reports suggest that loss of SMARCAL1 does not confer chemoresistance in BRCA1- or BRCA2-deficient cells (26,27).

Like SMARCAL1, CSB (Cockayne syndrome group B) is a DNA translocase that belongs to the SNF2 helicase family (12). First reported for its role in transcription-coupled nucleotide excision repair (TC-NER) (28,29), CSB has been implicated in DNA DSB repair (30–34) and the replication stress response (17,35,36). CSB possesses an intrinsic fork reversal activity *in vitro* (17), which is likely to be highly regulated *in vivo*. CSB is detected at ongoing replication forks in unperturbed cells (17,37,38) but becomes enriched at stalled forks upon replication stress (17). Similarly to SMARCAL1, loss of CSB leads to unrestrained fork progression upon mild replication stress (17). Loss of CSB also restores fork stability in cells deficient for BRCA1 or BRCA2 although this restoration does not confer chemoresistance (17). In contrast, loss of CSB leads to a further increase in chemosensitivity in cells deficient for BRCA1 or BRCA2, which is attributed to the role of CSB in promoting RAD52-dependent break-induced replication (BIR)-mediated restart of stalled forks (17). CSB also acts epistatically with RAD52 to promote mitotic DNA synthesis (MiDAS) upon replication stress (39). However, little is known about the epistatic relationship between CSB and other chromatin modelers at stalled forks.

In this report, we have discovered that CSB interacts with RPA and contains a RPA32 interaction motif in its N-terminal region. This interaction is necessary for promoting fork slowing and fork degradation in BRCA2-deficient cells. Our finding suggests that CSB competes with SMARCAL1 for RPA binding at stalled forks and acts non-redundantly with SMARCAL1 to restrain fork progression upon mild replication stress. Our work suggests that under the pathological condition lacking BRCA2, CSB and SMARCAL1 are engaged in distinct genetic pathways to control restart of stalled forks.

## Materials and methods

### Plasmids, siRNA and antibodies

CSB full length, CSB ATPase-dead W851R mutant, and various CSB deletion alleles that were fused to mCherry-LacR have previously been described (33). The previously reported Myc-tagged wild type CSB (pLPC-N-Myc-CSB) (40) was used as a template to generate, via site-directed mutagenesis, pLPC-N-Myc-CSB containing substitutions from R^176^Q^177^K^178^ to AAA (RQK-AAA) or from R^413^Q^414^K^415^ to AAA. Subsequently, the pLPC-N-Myc-CSB- R^176^Q^177^K^178^-AAA plasmid was digested with BglII and XhoI. The larger BglII-XhoI fragment was ligated with the smaller BglII-XhoI fragment derived from the previously reported pLPC-NMyc-CSB-T1031A (17), giving rise to pLPC-NMyc-CSB-R^176^Q^177^K^178^-AAA-T1031A. The cDNAs for RPA70 and RPA32, gifts from Alexey Bochkarev, University of Toronto, were subcloned into the retroviral expression vector pLPC-N-Myc (40) or pLPC-N-FH2 (41) (a kind gift from Titia de Lange, Rockefeller University). The GFP-based NHEJ reporter plasmid pEGFP-Pem1-Ad2 has previously been described (30,42). The GFP-based BIR reporter plasmid pBIR-GFP (43) (Addgene plasmid #49807) was a gift from Thanos Halazonetis.

Plasmids for recombinant bacterial protein expression were created as follows: wild type CSB was used as a template to generate via PCR the CSB fragment containing amino acids from 123 to 203 (CSB-F), which was subcloned into the bacterial expression vector pHis-parallel2 (44). The CSB-RQK-AAA mutant was used as a template to generate the CSB-F fragment carrying RQK-AAA mutations (CSB-F-AAA), which was subcloned via ligation-independent cloning (LIC) (45,46) and *Ssp*I restriction enzyme (R0132L, NEB) into pMCSG7 bacterial expression vector (Addgene) with a TEV-cleavable N-terminal 6X His-tag. The RPA32 construct spanning the region 210–270 (RPA32C) was cloned using LIC. Oligonucleotide primers used for cloning are available upon request. All plasmids were verified by Sanger sequencing.

siRNAs used were from Dharmacon: non-targeting siRNA (siControl; D-001206–14-05); siBRCA2 (D-003462–04) (17); siSMARCAL1 (D-013058–04-0002) (17); siPRIMPOL (GAGGAAAGCUGGACAUCGA) (47).

Antibodies used include: Biotin (1:100000; A150-109A, Bethyl Laboratories); Biotin (1:100000; 200-002-211, Jackson ImmunoResearch); 53BP1 (1:4000; 612522, BD Biosciences); BRCA2 (1:2000; 29450-1-AP, Proteintech); BrdU (1:50; 347580, BD Biosciences); BrdU (BU1/75[ICR1]) (1:400; NB500-169, Novus Biologicals); CSB (1:200; 553C5a, Fitzgerald); CSB (1:1000; A301-347A, Bethyl Laboratories); mCherry (1:10000; NBP2-25157, Novus Biologicals); HA-tag (1:500; #2367, Cell Signaling); MUS81 (1:2000; sc-47692, Santa Cruz); Myc (1:1000 for western, 1:2000 for IF; 9E10, Calbiochem); RPA70 (1:2000; 2267S, Cell Signaling); RPA32 (1:10000; NB100-332, Novus Biologicals); RPA32-pS4/pS8 (1:2000; A300-245A, Bethyl Laboratories); RPA32-pS33 (1:50000; A300-246A, Bethyl Laboratories), PRIMPOL (1:1000; 29824-1-AP, Proteintech); SMARCAL1 (1:50–100; sc-166209, Santa cruz); SMARCAL1 (1:2000; GTX109468, GeneTex); γ-tubulin (1:20000; GTU88, Sigma); α-tubulin (1:10000; T9026, Sigma).

### Cell culture, transfection, retroviral infection

U2OS (ATCC), U2OS CSB-KO (33), U2OS-265 CSB-KO (33), hTERT-RPE CSB-KO (30), HCT116 (ATCC), HCT116 CSB-KO (33), HEK293 (ATCC) and Phoenix (48) cells were grown in DMEM medium with 10% fetal bovine serum supplemented with non-essential amino acids, l-glutamine, 100 U/ml penicillin and 0.1 mg/ml streptomycin. Cell cultures were routinely fixed, stained with DAPI, and examined for mycoplasma contamination. Retroviral gene delivery was carried out as described (49,50) to generate stable cell lines. DNA and siRNA transfections were carried out with respective JetPrime^®^ transfection reagent (Polyplus) and Lipofectamine RNAiMAX (Invitrogen) according to their respective manufacturer's instructions.

### Proximity ligation (PLA) assays

PLA assays were performed using Duolink® PLA kit (Sigma) according to the manufacturer's instructions. Briefly, coverslips were blocked in Duolink® blocking solution for 30 min at 37°C and then incubated with primary antibody diluted in Duolink® antibody diluent overnight at 4°C. Following washes twice in wash buffer A [0.15 M NaCl, 10 mM Tris–HCl (pH7.4), 0.05% Tween-20] for 5 min, coverslips were incubated with anti-rabbit PLUS and anti-mouse MINUS PLA probes diluted in Duolink® antibody diluent for 1 h at 37°C. Subsequently, coverslips were washed twice in wash buffer A for 5 min, ligated for 30 min at 37°C, and then washed twice again in wash buffer A for 5 min. Amplification was performed using Duolink® *In Situ* Detection Reagents Green for 100 min at 37°C. Following amplification, coverslips were washed twice in wash buffer B (0.1 M NaCl, 0.2 M Tris) for 10 min and once in 0.1× wash buffer B for 1 min. Finally, coverslips were stained with DAPI (100 ng/ml in PBS). Cell images were recorded on a Zeiss Axioplan 2 microscope with a Hamamatsu C4742-95 camera and processed in Open Lab. PLA signals were quantified using ImageJ software (NIH).

### DNA fiber analysis

DNA fiber analysis was done essentially as described (17). For fork progression, cells were first incubated with 25 μM IdU (I7125, Sigma) for 30 min and then 250 μM CldU (C6891, Sigma) for 30 min in the presence of 50 μM HU. To evaluate the epistatic relationship between CSB and SMARCAL1 in fork progression, cells were first incubated with 25 μM IdU for 30 min and then 250 μM CldU for 60 min in the presence of 50 μM HU. For fork protection, cells were incubated first with 25 μM IdU for 20 min and then 250 μM CldU for 20 min prior to treatment with 4 mM HU for 5 h. For fork restart, cells were incubated with 25 μM IdU for 20 min, then treated with 4 mM HU for 4 h, followed by incubation with 250 μM CldU for 40 min. Following being spotted onto one end of a glass slide, cells were lysed in freshly made lysis buffer (50 mM EDTA pH 8.0, 200 mM Tris–HCl pH 7.5, 0.5% SDS) for 5 min and stretched onto the slide. Slides were fixed in freshly made methanol:acetic acid (3:1) for 20 min at -20°C and then allowed to air dry. Following incubation in freshly prepared 2.5 M HCl for 80 min, slides were washed three times in PBS and blocked with 5% BSA in PBS for 20 min at room temperature. Slides were then incubated with both rat anti-BrdU (1:800, NB500-169, Novus Biologicals) and mouse anti-BrdU (1:50, 347580, BD Sciences) antibodies prepared in 5% BSA in PBS for 1 h at 37°C. Subsequently, slides were washed three times in PBS and incubated with both Alexa-488 anti-rat (1:250, 712-545-153, Jackson ImmunoResearch) and Rhodamine anti-mouse (1:250, 715-295-151, Jackson ImmunoResearch) secondary antibodies for 1 h at room temperature. DNA fiber images were recorded on a Zeiss Axioplan 2 microscope with a Hamamatsu C4742-95 camera and processed in Open Lab. DNA fiber analysis was carried out with ImageJ software (NIH).

### S1 nuclease assays

S1 nuclease assays were done as described (36). To detect ssDNA gaps, cells were first incubated with 25 μM IdU for 20 min and then 250 μM CldU for 60 min in the presence of 50 μM HU. Prior to being spotted onto a glass slide, cells were pre-extracted by resuspending in 250 μl of cold CSK-100 buffer (10 mM MOPS pH 7.0, 100 mM NaCl, 3 mM MgCl_2_, 300 mM sucrose, 0.5% Triton X-100). Following incubation on ice for 10 min, cells were spun at 4000 rpm for 5 min at 4°C. Cell pellets were resuspended in 250 μl of freshly-made S1 nuclease buffer (30 mM NaAc pH 4.6, 10 mM ZnAc, 5% glycerol, 50 mM NaCl) and incubated in the presence or the absence of 20 units/ml of S1 nuclease (EN0321, ThermoFisher) for 30 min at 37°C. Following centrifugation at 4000 rpm for 5 min at 4°C, cell pellets were resuspended in 200 μl of cold PBS. The preparation and imaging of DNA fibers were done as described above.

### Immunofluorescence

Immunofluorescence (IF) was performed as described (17,30,40). To detect EdU, cells seeded on coverslips were treated with 10 μM EdU for 10 min prior to treatment with or without 4 mM HU. Following fixation, cells on coverslips were washed with PBS and then incubated with freshly prepared Click-iT reaction buffer (2 mM CuSO_4_, 10 μM biotin-PEG3-azide, 10 mM Ascorbic acid) for 10 min at room temperature. Coverslips were then washed in PBS twice, followed by regular IF as described (30,40). To detect HU-induced SMARCAL1 and RPA32 foci, cells were pre-extracted with cold CSK Buffer (10 mM PIPES pH 7.0, 100 mM NaCl, 300 mM Sucrose, 3 mM MgCl_2_, 0.7% Triton X-100) for 5 min prior to fixation. All cell images were recorded on a Zeiss Axioplan 2 microscope with a Hamamatsu C4742-95 camera and processed in Open Lab.

### GFP reporter assays and FACS analysis

GFP reporter assays were done as previously described (33,34). U2OS cells were first transfected with indicated siRNAs. Twenty-four hours later, cells were transfected with either pBIR-GFP or pEGFP-Pem1-Ad2, I-*SceI* and pCherry with a 4.5:4.5:1 ratio. Forty-eight hours later, cells were harvested, fixed and subjected to FACS analysis. Cherry expression was used as a transfection efficiency control. A total of 20000 events per cell line were scored for each independent experiment. FACS analysis was performed on a BD™ Accuri C6 Plus Flow Cytometer.

### Immunoprecipitation and immunoblotting

Immunoprecipitation was done as described (33). Immunoblotting was performed as described (34).

### Recombinant protein expression and purification

Expression plasmids for CSB_(123–203)_ (CSB-F), CSB_RQK-AAA(123–203)_ (CSB-F-AAA) and RPA32_(210–270)_ (RPA32C) were grown in Luria-Bertani (LB) broth at 37°C in *Escherichia coli* SoluBL21 (Genlantis) with expression induced at an OD_600_ of ∼0.7 with 0.5 mM IPTG for 3 h at 37°C. Bacterial cells were harvested by centrifugation, resuspended in lysis buffer (50 mM Tris–HCl pH8.0, 1 M NaCl, 2 mM β-mercaptoethanol, 10% (w/v) sucrose, 5% (v/v) glycerol, 0.1% (v/v) Triton X-100, 0.1% (v/v) NP-40, 0.5 mg/ml lysozyme), and lysed by sonication. Lysate was clarified by centrifugation and affinity purified by Ni-NTA IMAC resin (BioRad). Ni-NTA resin was washed with Ni-NTA wash buffer (50 mM Tris–HCl pH8.0, 400 mM NaCl, 10% (v/v) glycerol) and eluted in a stepwise gradient with 20, 40 and 400 mM imidazole in Ni-NTA wash buffer. CSB-F containing elutions were further purified using cation exchange chromatography, using a 5 ml HiTrap SP HP column (GE Healthcare), equilibrated with Q buffer (20 mM HEPES pH8.0, 2 mM β-mercaptoethanol, 10% (v/v) glycerol), and eluted over a gradient from 150 mM to 1 M NaCl. RPA32C containing elutions were further purified using anion exchange chromatography, using a 5 ml HiTrap Q HP column (GE Healthcare), equilibrated with Q buffer, and eluted over the same salt gradient as CSB. RPA32C was further purified through size exclusion chromatography (HiLoad 16/600 Superdex 75 pg (GE Healthcare) in S75 buffer (50 mM HEPES pH8.0, 400 mM NaCl, 10% (v/v) glycerol). Purified proteins were concentrated using a centrifugal concentrator (Cytiva) and were visualized by SDS-PAGE to assess purity. Proteins were stored at –80°C until use.

### Microscale thermophoresis

RPA32C was labeled using the His-tag Protein Labelling Kit RED-tris-NTA second generation (NanoTemper Technologies) according to the manufacturer's directions in the supplied labeling buffer, using 200 nM RPA32C (molar dye: protein ratio ∼ 1:4) at room temperature for 30 min protected from light. Unreacted dye was removed by centrifugation at 15000 × g for 10 min at 4°C. Labeled RPA32C was diluted to 100 nM with MST buffer (50 mM HEPES (pH 8.0), 400 mM NaCl, 10% glycerol, 0.05% Tween-20). Interacting proteins CSB-F, CSB-F-AAA and SMARCAL1 peptide (residues 5–30 [LTEEQRKKIEENRQKALARRAEKLLA], LifeTein, LLC) were serially diluted 2-fold in MST buffer, with the highest concentration starting at 40, 200 and 625 μM respectively. For the MST measurement, each protein sample was mixed with equal volumes of RPA32C, for a 1:1 ratio. Protein mixtures were incubated for 30 min at 37°C, then centrifuged for 10 min at 10000 × g. Samples were then loaded into Monolith Capillaries (NanoTemper Technologies) and placed in the Monolith instrument (NanoTemper Technologies) for MST measurements at 25°C. 40% LED power and Medium MST power were used in the data collection. Three independently pipetted replicates were analyzed from an MST-on time of 21 seconds using the MO control software (Version 2.6.2, NanoTemper Technologies). Data were fitted with a *K*_D_ fit using the MO control software (V 2.6.2, NanoTemper Technologies), and plotted in Prism (v. 9.5.1, GraphPad) as the mean ± standard deviation.

### AlphaFold modelling

The interaction of RPA32C and CSB-F model was carried out by ColabFold v1.5.2 using the AlphaFold2_mmseqs2 notebook (51,52). The AlphaFold2 predicted model was optimized both by reducing Ramachandran violations and steric clashes, as well as by increasing interactions between RPA32C and CSB-F models (primarily by altering sidechain rotamers), using the programs Coot 0.9.8.7 (53) and energy minimization in Maestro Version 12.9.123 (Schrödinger, LLC). Structural images were generated with the PyMol Molecular Graphics System, v2.5.0 (Schrödinger, LLC). Interaction analysis including hydrogen bonds and salt bridges was carried out using PDBePISA (www.ebi.ac.uk/pdbe/pisa/).

### Cell viability and clonogenic survival assays

Cell viability assays were done as described (54). Briefly, cells were seeded in 24-well plates at 30 000 cells per well. Twenty-four hours later, cells were transfected with indicated siRNAs. Forty-eight hours post transfection, cells were harvested, counted, and seeded in 96-well plates at 5000 cells per well. Seventy-two hours later, cells were stained with 0.5% crystal violet in methanol. The optical density of crystal violet staining was measured at 584 nm using BioTek Cytation 5 Cell Imaging Multimode Reader. Clonogenic survival assays were done as described (30).

### Statistical analysis

A Student's two-tailed unpaired *t*-test was used to derive all *P* values except for where specified.

## Results

### CSB interacts with RPA32 at stalled replication forks

**Figure 1.**
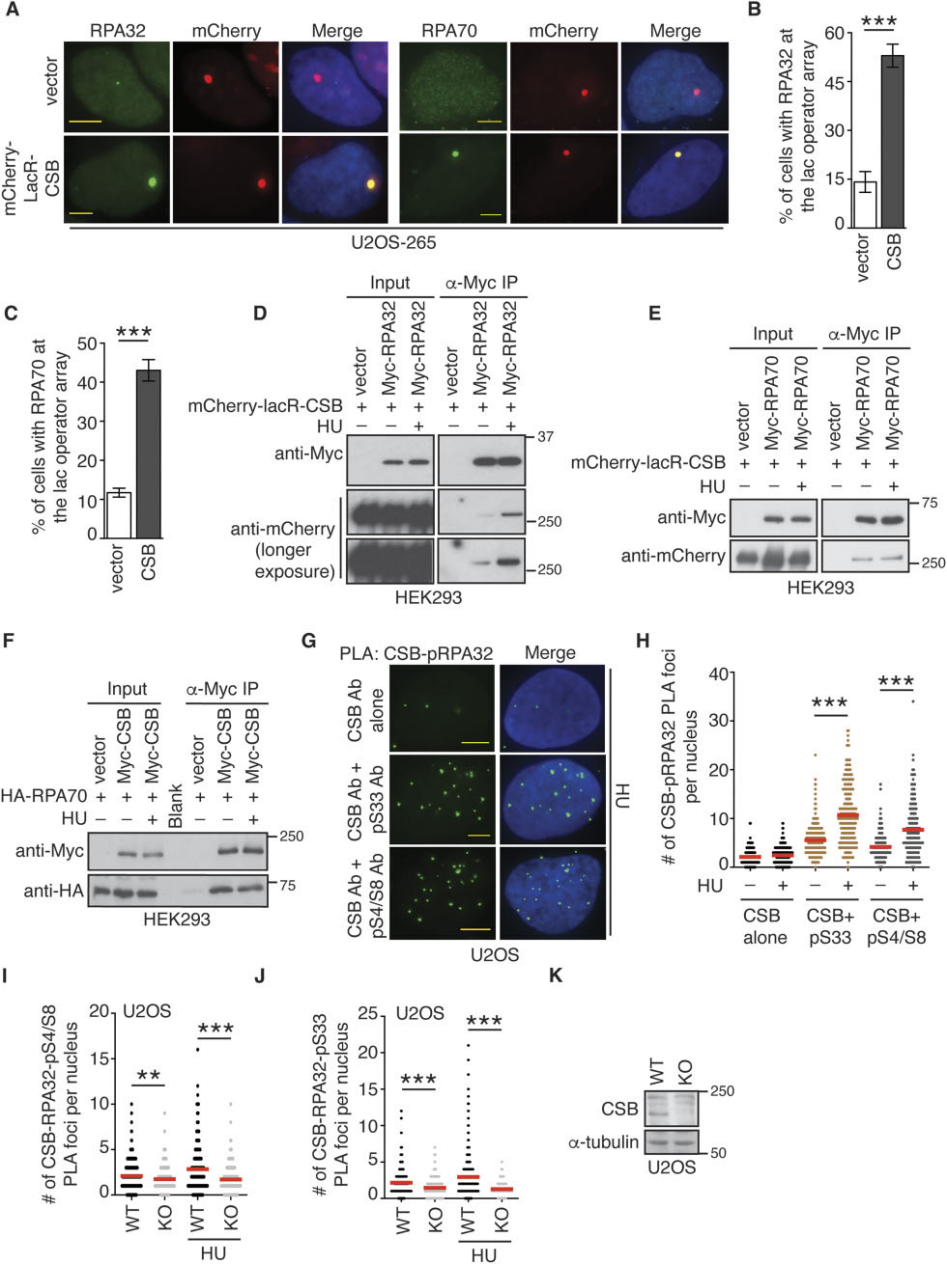
CSB interacts with RPA at stalled replication forks. (**A**) Representative images of U2OS-265 CSB-KO cells expressing the vector alone or mCherry-LacR-CSB. Immunostaining was done with an either anti-RPA32 or anti-RPA70 antibody (green). Cell nuclei were stained with DAPI in blue in this and subsequent figures. Scale bars in this and subsequent panels: 5 μm. (**B**) Quantification of the percentage of cells exhibiting RPA32 accumulated at the lac operator array from (A). At least 100 cells expressing mCherry-LacR-CSB were scored per condition in a blind manner. Standard deviations (SDs) from three independent experiments are indicated in this and 1C. ***P< 0.001. (**C**) Quantification of the percentage of cells exhibiting RPA70 accumulated at the lac operator array from (A). Scoring was done as described in (B). ***P< 0.001. (**D**) Coimmunoprecipitation with anti-Myc antibody in HEK293 cells transfected with mCherry-LacR-CSB in conjunction with the vector alone or Myc-RPA32 in the presence or absence of HU. For HU treatment, cells were collected 4 h post treatment with 4 mM HU. Immunoblotting was performed with anti-Myc and anti-mCherry antibodies. (**E**) Coimmunoprecipitation with anti-Myc antibody in HEK293 cells transfected with mCherry-LacR-CSB in conjunction with the vector alone or Myc-RPA70 in the presence or the absence of HU. Immunoblotting was performed with anti-Myc and anti-mCherry antibodies. (**F**) Coimmunoprecipitation with anti-Myc antibody in HEK293 cells transfected with HA-RPA70 in conjunction with the vector alone or Myc-CSB in the presence or absence of HU. Immunoblotting was performed with anti-Myc and anti-HA antibodies. (**G**) Representative images of PLA between CSB and either RPA32-pS33 or RPA32-pS4/S8 in U2OS cells treated with or without HU. For HU treatment, cells were fixed 4 h post treatment with 4 mM HU. (**H**) Quantification of PLA between CSB and either RPA32-pS33 or RPA32-pS4/S8 from (G). The respective number of cells analyzed for CSB alone (–HU), CSB alone (+HU), CSB + RPA32-pS33 (–HU), CSB + RPA32-pS33 (+HU), CSB + RPA-pS4/S8 (–HU) and CSB + RPA-pS4/S8 (+HU) were 160, 269, 291, 269, 169 and 169. Data from single experiments are represented as scatter plot graphs with the mean indicated in this, (I) and (J) panels. The *P*-value was determined using a non-parametric Mann-Whitney rank-sum *t*-test in this, (I) and (J) panels. ***P< 0.001. (**I**) Quantification of PLA between CSB and RPA32-pS4/S8 in both U2OS WT and CSB-knockout (KO) cells treated with or without HU. The respective number of cells analyzed for U2OS WT (–HU), U2OS WT (+HU), U2OS CSB-KO (–HU), and U2OS CSB-KO (+HU) were 297, 300, 245, 277. (**J**) Quantification of PLA between CSB and RPA32-pS33 in both U2OS WT and CSB-KO cells treated with or without HU. The respective number of cells analyzed for U2OS WT (–HU), U2OS WT (+HU), U2OS CSB-KO (–HU) and U2OS CSB-KO (+HU) were 263, 281, 272, 271. (**K**) Western analysis of U2OS WT and CSB-KO cells. Immunoblotting was performed with anti-CSB and anti-α-tubulin antibodies. The α-tubulin blot was used as a loading control.

Uncoupling of stalled forks leads to accumulation of ssDNA, which is rapidly bound by RPA, a ssDNA binding protein. RPA has been reported to both interact with the fork remodeler SMARCAL1 (15) and regulate fork reversal activity of both SMARCAL1 and ZRANB3 (55). We have recently reported that CSB is associated with stalled forks to promote fork reversal (17). Therefore, we asked whether CSB might interact with RPA. To address this question, we employed our previously established reporter osteosarcoma cell line U2OS-265 CSB-KO (33), which contains the 256 copy lac operator array integrated into a single site on chromosome 1p3.6. This cell line allows for analysis of protein-protein interactions with a bait protein fused to mCherry-LacR. Using this cell line, we observed an interaction between mCherry-LacR-CSB and endogenous RPA32 or RPA70 at the lac operator array (Figure 1A–C). To substantiate this interaction, we performed coimmunoprecipitation in HEK293 cells overexpressing mCherry-LacR-CSB in combination with either Myc-RPA70 or Myc-RPA32. Both Myc-RPA70 and Myc-RPA32 brought down mCherry-LacR-CSB (Figure 1D and E). In a reciprocal immunoprecipitation, HA-RPA70 was also brought down by Myc-CSB (Figure 1F), supporting the notion that CSB interacts with RPA. Interestingly, we observed that treatment with HU stimulated the interaction of mCherry-LacR-CSB with Myc-RPA32 but not Myc-RPA70 (Figure 1D and E). As RPA is a trimeric complex, this discrepancy is unexpected and requires future investigation. Nevertheless, these results suggest that it is likely that CSB interacts with RPA in a replication stress-induced manner.

We have previously reported that the level of exogenously expressed CSB is higher than that of endogenous CSB (56). Therefore, we asked whether endogenous CSB interacts with RPA. However, co-immunoprecipitation with an antibody against endogenous CSB failed to bring down endogenous RPA70 (Supplementary Figure S1A). To address the possibility that the CSB-RPA interaction might be transient or of low abundance, we turned to a proximity ligation-based assay (PLA) that allows measurement of protein interactions *in situ*. Analysis of PLA assays revealed that treatment with HU in U2OS cells led to a significant increase in the number of PLA foci between endogenous CSB and RPA32-pS33 or RPA32-pS4/S8 (Figure 1G and H), both of which are markers for replication stress. This increase was abrogated in U2OS CSB-KO cells (Figure 1I–K), suggesting that CSB interacts with RPA32 at stalled forks and that this interaction is likely to be transient or low in abundance. We also observed the PLA foci between CSB and RPA32-pS33 or RPA32-pS4/S8 in untreated U2OS cells (Figure 1H), in agreement with previous findings that CSB is associated with ongoing replication forks (17,38).

We have previously reported that CSB is recruited to sites of DSBs (30,33), which can arise from MUS81-mediated processing of stalled forks (57). To investigate whether DSBs at stalled forks mediate CSB-RPA32-pS4/S8 PLA foci formation, we knocked down MUS81 in U2OS cells. Analysis of PLA assays revealed that depletion of MUS81 did not impair HU-induced formation of PLA foci between CSB and RPA32-pS4/S8 (Supplementary Figure S1B and S1C), suggesting that the interaction of CSB with RPA32 at stalled forks is unlikely to be mediated by DSBs arising from MUS81-mediated processing of stalled forks.

### CSB interacts with RPA through its N-terminal region

**Figure 2.**
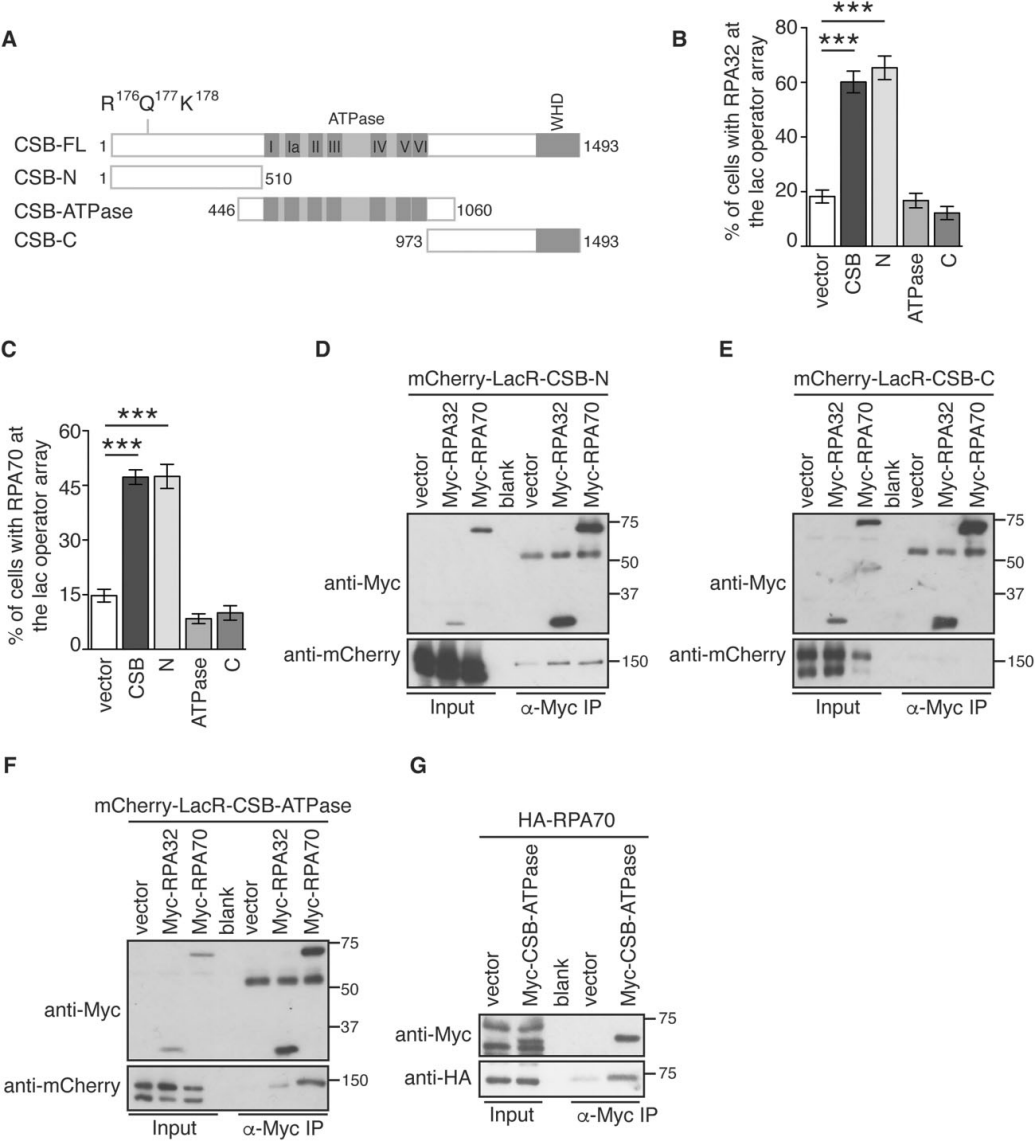
The N-terminal region of CSB interacts with RPA. (**A**) Schematic diagram of CSB. (**B**) Quantification of the percentage of cells exhibiting RPA32 accumulated at the lac operator array. U2OS-265 CSB-KO cells were transfected with various CSB alleles as indicated. At least 100 cells expressing various mCherry-LacR-CSB alleles were scored per condition in a blind manner. Standard deviations (SDs) from three independent experiments are indicated in this and (C). ***P< 0.001. (**C**) Quantification of the percentage of cells exhibiting RPA70 accumulated at the lac operator array. U2OS-265 CSB-KO cells were transfected with various CSB alleles as indicated. Scoring was done as described in (B). ***P< 0.001. (**D**) Coimmunoprecipitation with anti-Myc antibody in HEK293 cells transfected with mCherry-LacR-CSB-N in conjunction with the vector alone, Myc-RPA32, or Myc-RPA70. Immunoblotting was performed with anti-Myc and anti-mCherry antibodies. (**E**) Coimmunoprecipitation with anti-Myc antibody in HEK293 cells transfected with mCherry-LacR-CSB-C in conjunction with the vector alone, Myc-RPA32, or Myc-RPA70. Immunoblotting was performed with anti-Myc and anti-mCherry antibodies. (**F**) Coimmunoprecipitation with anti-Myc antibody in HEK293 cells transfected with mCherry-LacR-CSB-ATPase in conjunction with the vector alone, Myc-RPA32 or Myc-RPA70. Immunoblotting was performed with anti-Myc and anti-mCherry antibodies. (**G**) Coimmunoprecipitation with anti-Myc antibody in HEK293 cells transfected with HA-RPA70 in conjunction with the vector alone or Myc-CSB carrying the ATPase domain alone (Myc-CSB-ATPase). Immunoblotting was performed with anti-Myc and anti-HA antibodies.

To map domains of CSB that interact with RPA, we employed previously reported mCherry-LacR-CSB deletion alleles (33) containing the N-terminal region alone (CSB-N), the ATPase domain alone (CSB-ATPase), or the C-terminal region alone (CSB-C) (Figure 2A). We first examined their ability to interact with RPA70 and RPA32 at the lac operator array in U2OS-265 CSB-KO cells. When expressed in the reporter U2OS-265 CSB-KO cells, mCherry-LacR-CSB-N was able to recruit endogenous RPA32 or RPA70 to the lac operator array, indistinguishably from mCherry-LacR-CSB full length (Figure 2B and C, Supplementary Figure S2A and S2B). In contrast, neither mCherry-LacR-CSB-ATPase nor mCherry-LacR-CSB-C interacted with RPA32 or RPA70 at the lac operator array (Figure 2B-C). To further investigate the ability of these various CSB deletion alleles to interact with RPA, we performed coimmunoprecipitation (co-IP) analyses in HEK293 cells overexpressing each of these CSB deletion alleles in combination with either Myc-RPA70 or Myc-RPA32. Myc-RPA32 and Myc-RPA70 were found to bring down mCherry-LacR-CSB-N but not mCherry-LacR-CSB-C (Figure 2D and E). Interestingly, both Myc-RPA32 and Myc-RPA70 also brought down mCherry-LacR-CSB-ATPase although Myc-RPA70 exhibited a stronger ability to pull down mCherry-LacR-CSB-ATPase than Myc-RPA32 (Figure 2F). The interaction between the ATPase domain of CSB and RPA70 was also observed in a reciprocal coimmunoprecipitation. HA-RPA70 was readily brought down by Myc-CSB-ATPase (Figure 2G). This finding was in contrast to a lack of interaction observed between mCherry-LacR-CSB-ATPase and either RPA70 or RPA32 in the reporter U2OS-265-CSB-KO cells (Figure 2B and C). This discrepancy is likely due to the difference in the two methods, coimmunoprecipitation vs the reporter cell line. Nevertheless, these results altogether suggest that CSB interacts with RPA through both its N-terminal region and its ATPase domain.

### The N-terminal region of CSB directly interacts with the C-terminal domain of RPA32

**Figure 3.**
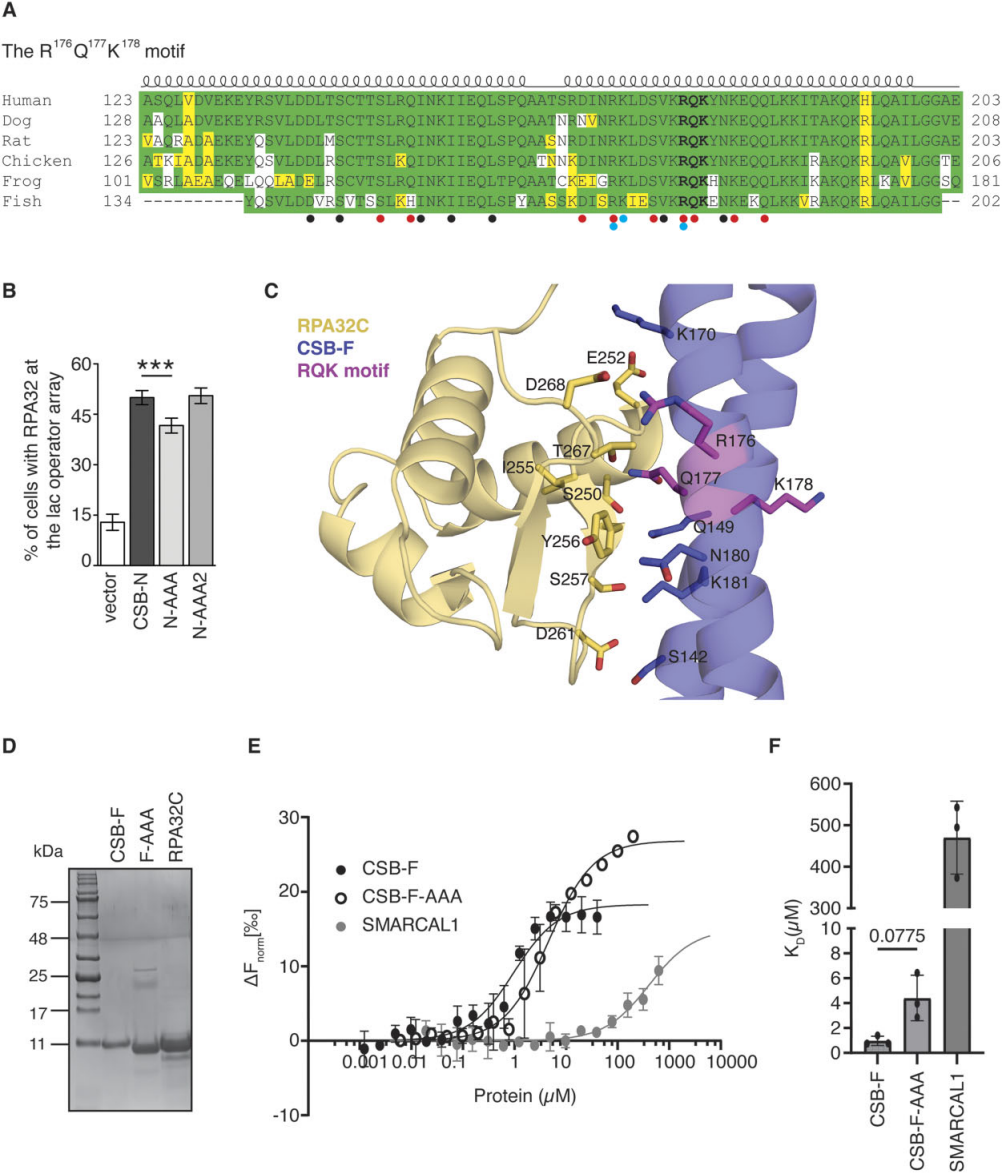
CSB contains a highly conserved N-terminal RPA32-interacting motif. (**A**) Sequence alignment of the RPA32C-binding region of CSB with selective vertebrate orthologs. The R^176^Q^177^K^178^ motif is indicated in bold. Compared to human CSB, identical amino acids, similar amino acids (charged RHKDE, polar uncharged STNQY, hydrophobic AVILMFWCPG), and non-similar amino acids are highlighted in green, yellow, and white, respectively. Cartoon of the secondary structure is depicted above the sequences. Residues shown to interact between RPA32 and CSB in the optimized AlphaFold2 model are depicted with black circles, with red and blue circles additionally denoting hydrogen bonds and salt bridges, respectively. Accession numbers are: human ERCC-6, NP_001333369; dog ERCC-6 XP_534944.2; rat ERCC-6 NP_001100766.1; chicken ERCC-6, XP_421656.3; frog ERCC-6 NP_001361595.1; fish ERCC-6, XP_005815483.2. (**B**) Quantification of the percentage of cells exhibiting RPA32 accumulated at the lac operator array. At least 100 cells expressing various mCherry-LacR-CSB-N alleles as indicated were scored per condition in a blind manner. SDs from three independent experiments are indicated. ***P< 0.001. (**C**) Optimized AlphaFold model of RPA32C (yellow) in complex with CSB-F (blue). The R^176^Q^177^K^178^ motif is highlighted in magenta, with some interacting residues shown as sticks, where red represents oxygen and blue nitrogen. (**D**) Coomassie staining of purified recombinant CSB containing amino acids from 123 to 203 (CSB-F), CSB-F carrying R^176^Q^177^K^178^-AAA mutations, and RPA32C from bacterial cells. (**E**) MST binding curves of CSB-F, CSB-F-AAA, and SMARCAL1 binding to RPA32C. Data are plotted as the change in normalized fluorescence (Δ*F*_norm_) of RPA32C against concentration of the ligands, CSB-F, CSB-F-AAA and SMARCAL1, required for calculating the dissociation constant, K_D_. Three independent measurements were completed for each experiment, with data plotted as the mean ± standard deviation. (**F**) *K*_D_ values for CSB-F, CSB-F-AAA and SMARCAL1 bound to RPA32C. Data are plotted as the mean ± standard deviation with three independent measurements. *P*-value was calculated using a two-tailed Welch's *t*-test using Prism V 9.5.1 (GraphPad).

The C-terminal winged helix domain of RPA32 (RPA32C) interacts directly with several DNA replication and repair proteins including SMARCAL1, RAD52, XPA and UNG2 (14,58). It has been reported that both SMARCAL1 and RAD52 contain a conserved RQK motif in their respective RPA32 binding region and that mutating RQK to AAA reduces their interaction with RPA32 (14,59). Sequence analysis revealed that the N-terminal region of CSB contains two RQK motifs, R^176^Q^177^K^178^ and R^413^Q^414^K^415^. The R^176^Q^177^K^178^ motif is more highly conserved among vetebrates compared to the R^413^Q^414^K^415^ motif (Figure 3A and Supplementary Figure S3A). According to the CSB Alphafold structure (AF-Q03468) (39), while the R^413^Q^414^K^415^ motif is found within a disordered region, the R^176^Q^177^K^178^ motif is located in a region that is contained within an α helix, as are the equivalent regions shown to be important in the structural studies of the binding of UNG2 and SMARCAL1 to RPA32C (58,60). Nevertheless, we asked whether either of these RQK motifs mediates the interaction of CSB with RPA32. To address this question, we generated mCherry-LacR-CSB-N mutants carrying amino acid substitutions either from R^176^Q^177^K^178^ to three alanines (AAA) or from R^413^Q^414^K^415^ to three alanines (AAA). The CSB-N-R^176^Q^177^K^178^-AAA and CSB-N-R^413^Q^414^K^415^-AAA mutants were referred to as CSB-N-AAA and CSB-N-AAA-2, respectively. Using the reporter U2OS-265 CSB-KO cells, we found that compared to mCherry-LacR-CSB-N, mCherry-LacR-CSB-N-AAA but not mCherry-LacR-CSB-N-AAA-2 exhibited a mild but significant defect in recruiting RPA32 to the lac operator (Figure 3B and Supplementary Figure S3B). These results suggest that the R^176^Q^177^K^178^ motif is necessary for an efficient interaction between the N-terminal region of CSB and RPA32. We observed that mCherry-LacR-CSB full length carrying R^176^Q^177^K^178^-AAA mutations coimmunoprecipitated with Myc-RPA70 (Supplementary Figure S3C), in agreement with the notion that CSB interacts with RPA through more than one region.

To provide further context on the interaction between CSB and RPA32, we next turned to computational modeling. Previous structures of RPA32 with UNG2 and SMARCAL1 have shown that binding to RPA32 is accomplished primarily via interactions with an α-helical face. On the other hand, a model of SV40 T-antigen has been shown to interact with a similar face of RPA32C through a series of extended loops (61). As the AlphaFold prediction of CSB (AF-Q03468) shows that the helix containing the R^176^Q^177^K^178^ motif forms a coiled-coil domain with a helix directly upstream, we decided to carry out computational and subsequent *in vitro* binding experiments with the region 123–203 (CSB-F) encasing portions of both helices in order to capture potential interactions with RPA32C.

The computational modelling of RPA32C and CSB-F revealed that R^176^Q^177^K^178^ of CSB is partly engaged in forming a binding interface with RPA32C (Figure 3C and Supplementary Figure S4A). Q^177^ of CSB is predicted as the primary point of interaction with RPA32, through hydrogen bonding with the side chain of T^267^ and the main-chain carbonyl of I^255^ of RPA32C (Figure 3C). It is possible that R^176^ of CSB also contributes to the interaction with E^270^ and/or D^268^ of RPA32C. The pLDDT score of the original AlphaFold2 model indicated a low confidence in prediction accuracy of C-terminal residues D^268^-E^270^ in RPA32C (Supplementary Figure S4B), suggesting that this is a flexible region and could accommodate these interactions with R^176^ of CSB. Alternatively, energy minimization to optimize geometry of the AlphaFold2 model allows the R^176^ sidechain rotamer to form salt bridges with D^268^ of RPA32C (Figure 3C). Numerous residues in RPA32C such as S^250^, E^252^, Y^256^, S^257^, D^261^ and T^267^, which have previously been shown to be important for the interactions with UNG2, SMARCAL1 or SV40 T-antigen (58,60,61), are shown in the optimized AlphaFold2 model to interact with CSB (Figure 3C). Comparison of the optimized AlphaFold2 model and experimental structures of UNG2 and SMARCAL1 bound to RPA32 revealed a conserved protein binding interface on RPA32C (Supplementary Figure S4C). RPA32 structures bound to UNG2 and SMARCAL1 aligned to the optimized RPA32C AlphaFold2 model with an RMSD of 0.51 and 0.47 Å, respectively, showing the RPA32C binding interface is composed primarily of the C-terminal β-sheet of the winged helix domain (Supplementary Figure S4C). The conserved RQK motif found in RPA32 binding partners CSB-F, SMARCAL1 and UNG2 contributes to the interaction with RPA32, although not all amino acids of the motif are involved (Supplementary Figure S4D) and is dependent upon the protein binding partner. Interestingly, while previously determined structures show binding of RPA32 to a single helix, our AlphaFold2 model shows additional interactions of RPA32C with an upstream helix of CSB (Figure 3C, Supplementary Figure S4E).

To further investigate if CSB directly interacts with RPA32, we produced bacterial-expressed recombinant RPA32C, the CSB fragment (CSB-F) containing amino acids from 123 to 203, which includes the R^176^Q^177^K^178^ motif, as well as CSB-F carrying R^176^Q^177^K^178^-AAA mutations (CSB-F-AAA) (Figure 3D). Analysis of microscale thermophoresis measurements (MST) revealed a direct binding of RPA32C by CSB-F with a *K*_D_ of 0.94 ± 0.37 μM (Figure 3E and F, Supplementary Figure S5A and S5B). The CSB-F-AAA mutant exhibited an ∼5-fold reduction in affinity to RPA32C with a *K*_D_ of 4.41 ± 1.83 μM (Figure 3E and F), suggesting that the conserved R^176^Q^177^K^178^ motif is important for the stability of the interaction between CSB-F and RPA32C. It has been reported that a 26-amino acid peptide derived from SMARCAL1 (residues 5–30) binds to RPA32C with a *K*_D_ of 2.9 ± 0.1 μM through analysis of isothermal titration calorimetry (ITC) (60). To investigate how CSB’s binding to RPA32C is comparable to SMARCAL1’s binding to RPA32C, we measured, via MST analysis, the binding of this 26-amino acid SMARCAL1 peptide to RPA32C. Compared to CSB-F, SMARCAL1 peptide exhibited very weak binding to RPA32C with a *K*_D_ of 470.3 ± 87.64 μM (Figure 3F and Supplementary Figure S5C), which is in contrast to the previously-reported *K*_D_ of 2.9 ± 0.1 μM. This discrepancy is likely due to the difference in experimental conditions, ITC versus MST and RPA32C constructs used (RPA32C (201–272) for ITC versus RPA32C (210–270) for MST). Taken together, these results demonstrate that the N-terminal region of CSB directly binds the C-terminal domain of RPA32, with the conserved R^176^Q^177^K^178^ motif of CSB contributing to the stability of the interaction.

### An efficient association of CSB with stalled forks is dependent upon its R^176^Q^177^K^178^ motif

**Figure 4.**
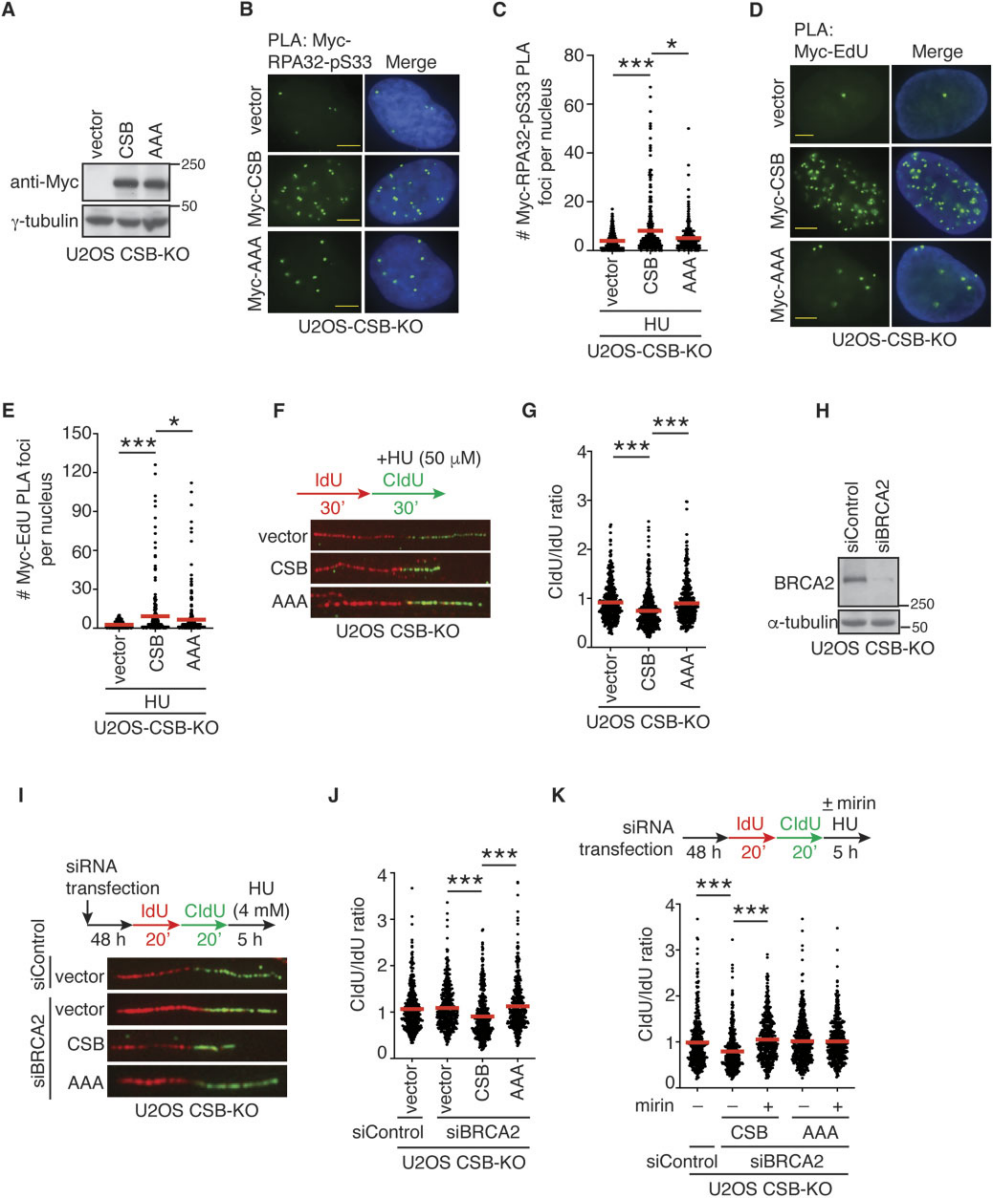
CSB relies on the R^176^Q^177^K^178^ motif to promote fork slowing and fork degradation in BRCA2-deficient cells. (**A**) Western analysis of U2OS CSB-KO cells expressing the vector alone, Myc-CSB, or Myc-CSB carrying R^176^Q^177^K^178^-AAA mutations (AAA). Immunoblotting was done with anti-Myc and anti-γ-tubulin antibodies. The γ-tubulin blot was used as a loading control in this and subsequent figures. (**B**) Representative images of PLA foci formation between Myc staining and RPA32-pS33 in U2OS CSB-KO expressing the vector alone, Myc-CSB or Myc-CSB carrying R^176^Q^177^K^178^-AAA mutations (AAA). (**C**) Quantification of PLA between anti-Myc and RPA-pS33 in HU-treated U2OS CSB-KO cells expressing the vector alone, Myc-CSB, or Myc-CSB carrying R^176^Q^177^K^178^-AAA mutations (AAA). A total of 321–329 cells were scored per condition in a blind manner. Data from single experiments are represented as scatter plot graphs with the mean indicated in this, (E), (G), (J) and (K) panels. The *P*-value was determined using a non-parametric Mann–Whitney rank-sum *t*-test in this, (E), (G), (J) and (K) panels. *P< 0.05; ***P< 0.001. (**D**) Representative images of PLA foci formation between Myc staining and EdU in U2OS CSB-KO expressing the vector alone, Myc-CSB or Myc-CSB carrying R^176^Q^177^K^178^-AAA mutations (AAA). (**E**) Quantification of PLA between anti-Myc and EdU in HU-treated U2OS CSB-KO cells expressing the vector alone, Myc-CSB, or Myc-CSB carrying R^176^Q^177^K^178^-AAA mutations (AAA). A total of 252–276 cells were scored per condition in a blind manner. *P< 0.05; ***P< 0.001. (**F**) Representative images of DNA fibers from U2OS CSB-KO expressing the vector alone, Myc-CSB or Myc-CSB carrying R^176^Q^177^K^178^-AAA mutations (AAA) that were first labeled with IdU (red) and then labeled with CldU (green) in the presence of 50 μM HU. (**G**) Quantification of the CldU/IdU ratio from U2OS CSB-KO cells expressing the vector alone, Myc-CSB, or Myc-CSB carrying R^176^Q^177^K^178^-AAA mutations (AAA). A total of 417–447 fibers per condition were analyzed. ***P< 0.001. (**E**) Quantification of the percentage of stalled forks from U2OS CSB-KO cells expressing the vector alone, Myc-CSB or Myc-CSB carrying R^176^Q^177^K^178^-AAA mutations (AAA). A total of 315–363 fibres per condition were scored in a blind manner. SDs from three independent experiments are shown. ***P< 0.001. (**H**) Western analysis of U2OS CSB-KO cells transfected with siControl or siBRCA2. Immunoblotting was performed with anti-BRCA2 and γ-tubulin antibodies. (**I**) Representative images of DNA fibers from U2OS CSB-KO expressing the vector alone, Myc-CSB or Myc-CSB-AAA. Following transfection with indicated siRNA, cells were incubated first with IdU (red) and then with CldU (green), followed by treatment with 4 mM HU for 5 h. (**J**) Quantification of the CldU/IdU ratio from siBRCA2-transfected U2OS CSB-KO cells expressing the vector alone, Myc-CSB or Myc-CSB carrying R^176^Q^177^K^178^-AAA mutations (AAA). A total of 426–461 fibers per condition were analyzed. ***P< 0.001. (**K**) Quantification of the CldU/IdU ratio from siBRCA2-transfected U2OS CSB-KO cells expressing Myc-CSB or Myc-CSB carrying R^176^Q^177^K^178^-AAA mutations (AAA). Following the CldU labeling, cells were treated with HU in the presence or absence of 50 μM mirin for 5 h. A total of 424–461 fibers per condition were analyzed. ***P< 0.001.

To investigate if CSB relies on its R^176^Q^177^K^178^ motif for its function at stalled forks, we first generated U2OS CSB-KO cells stably expressing the vector alone, Myc-CSB or Myc-CSB-R^176^Q^177^K^178^-AAA (Figure 4A). Analysis of PLA assays revealed that the R^176^Q^177^K^178^-AAA mutations impaired the ability of Myc-CSB to form PLA foci with RPA32-pS33 in response to treatment with HU in U2OS CSB-KO cells (Figure 4B and C). The R^176^Q^177^K^178^-AAA mutations also impaired the ability of Myc-CSB to form PLA foci with RPA32-pS33 in response to treatment with HU in our previously reported hTERT-RPE-CSB-KO cells (30) (Supplementary Figure S6A), suggesting that the effect of R^176^Q^177^K^178^-AAA mutations on CSB’s interaction with RPA at stalled forks is not specific to U2OS cells. To further substantiate whether the R^176^Q^177^K^178^-AAA mutations affect CSB’s association with stalled forks, we pulse-labeled the aforementioned cells with EdU prior to treatment with HU. Analysis of PLA assays revealed that the R^176^Q^177^K^178^-AAA mutations impaired Myc-CSB-EdU PLA foci formation in response to treatment with HU in both U2OS CSB-KO and hTERT-RPE CSB-KO cells (Figure 4D and E; Supplementary Figure S6B). The impaired but not lack of association of the Myc-CSB-R^176^Q^177^K^178^-AAA mutant with stalled forks is likely due to the ability of CSB to interact with RPA through region(s) other than CSB’s R^176^Q^177^K^178^ motif since we have shown that CSB is likely to interact with RPA70 through its ATPase domain (Figure 2F and G). These results altogether suggest that the CSB-RPA32 interaction is necessary for efficient association of CSB with stalled forks, in agreement with our *in vitro* finding that the R^176^Q^177^K^178^ motif contributes to the stability of the CSB–RPA32 interaction.

### CSB relies on its R^176^Q^177^K^178^ motif to restrain fork progression

We have previously reported that CSB restrains fork progression upon exposure to mild replication stress (17). To examine whether CSB relies on the R^176^Q^177^K^178^ motif to restrain fork progression, U2OS CSB-KO cells stably expressing the vector alone, Myc-CSB or Myc-CSB-R^176^Q^177^K^178^-AAA were first labeled with IdU for 30 min and then with CldU for 30 min in the presence of 50 μM HU. DNA fiber analysis revealed that while overexpression of Myc-CSB led to a reduction in the ratio of CldU/IdU in U2OS CSB-KO cells, overexpression of Myc-CSB-R^176^Q^177^K^178^-AAA failed to do so (Figure 4F and G). The inability of Myc-CSB-R^176^Q^177^K^178^-AAA to promote a reduction in the ratio of CldU/IdU in the presence of 50 μM HU was also observed in our previously-reported HCT116 CSB-KO cells (33) (Supplementary Figure S6C). The inability of Myc-CSB-R^176^Q^177^K^178^-AAA to restrain fork progression in the presence of 50 μM HU was indistinguishable from that of Myc-CSB carrying a previously reported ATPase-dead W851R mutation (Supplementary Figure S6D). These results altogether suggest that like CSB’s ATPase activity, the R^176^Q^177^K^178^ motif of CSB is essential for restraining fork progression in response to mild replication stress.

### CSB relies on its R^176^Q^177^K^178^ motif to promote fork degradation in BRCA2-depleted cells

We have previously reported that CSB promotes MRE11-dependent fork degradation in BRCA1/BRCA2 deficient cells (17). To investigate whether CSB relies on the R^176^Q^177^K^178^ motif to promote fork degradation in BRCA-deficient cells, we first knocked down BRCA2 in U2OS CSB-KO cells (Figure 4H), followed by transfection with the vector alone, Myc-CSB or Myc-CSB-R^176^Q^177^K^178^-AAA. Subsequently, these cells were labeled with IdU for 20 min and then with CldU for 20 min, followed by treatment with 4 mM HU for 5 hours. DNA fiber analysis revealed that depletion of BRCA2 led to a reduction in the ratio of CldU/IdU in U2OS CSB-KO expressing Myc-CSB (Figure 4I and J). This reduction was not observed in U2OS CSB-KO cells expressing either the vector alone or Myc-CSB-R^176^Q^177^K^178^-AAA (Figure 4I and J). The inability of Myc-CSB-R^176^Q^177^K^178^-AAA to suppress the ratio of CldU/IdU was also observed in BRCA2-depleted HCT116 CSB-KO cells (Supplementary Figure S6E). These results altogether suggest that CSB is dependent upon its R^176^Q^177^K^178^ motif to promote fork degradation in BRCA2-deficient cells.

It has been well described that MRE11 mediates fork degradation in BRCA2-depleted cells (22–24). To investigate whether CSB relies on its R^176^Q^177^K^178^ motif to promote MRE11-dependent fork degradation, BRCA2-depleted U2OS CSB-KO cells expressing Myc-CSB or Myc-CSB-R^176^Q^177^K^178^-AAA were labeled with IdU for 20 min and then with CldU for 20 min, followed by treatment with 4 mM HU in the presence or absence of MRE11 inhibitor mirin for 5 h. Treatment with mirin restored the ratio of CldU/IdU in BRCA2-depleted U2OS CSB-KO cells expressing Myc-CSB (Figure 4K), in agreement with previous findings. Treatment with mirin had little effect on the ratio of CldU/IdU in BRCA2-depleted U2OS CSB-KO cells expressing Myc-CSB-R^176^Q^177^K^178^-AAA (Figure 4K), suggesting that the R^176^Q^177^K^178^ motif of CSB is necessary for MRE11-dependent fork degradation in BRCA2-deficient cells.

### CSB’s R^176^Q^177^K^178^ motif is epistatic to CSB’s phosphorylation on T1031 at stalled forks

We have previously reported that CSB is phosphorylated by CDK on T1031 and that this phosphorylation mediates CSB’s recruitment to stalled forks as well as CSB’s ability to promote MRE11-dependent fork degradation in BRCA-deficient cells (17). To investigate whether CSB’s R^176^Q^177^K^178^ motif is epistatic to CSB’s phosphorylation on T1031 at stalled forks, we generated Myc-CSB carrying combined mutations of R^176^Q^177^K^178^-AAA and T1031A. Analysis of PLA assays revealed that Myc-CSB-R^176^Q^177^K^178^-AAA-T1031A was defective in its association with stalled forks, indistinguishably from Myc-CSB-R^176^Q^177^K^178^-AAA or Myc-CSB-T1031A (Supplementary Figure S7A). In addition, DNA fiber assays revealed that Myc-CSB-R^176^Q^177^K^178^-AAA-T1031A failed to promote fork slowing in U2OS CSB-KO cells, as well as fork degradation in BRCA2-depleted U2OS CSB-KO cells, indistinguishably from Myc-CSB-R^176^Q^177^K^178^-AAA or Myc-CSB-T1031A (Supplementary Figure S7B and S7C). These results altogether suggest that CSB’s R^176^Q^177^K^178^ motif and CSB’s phosphorylation on T1031 function in the same pathway at stalled forks.

### The R^176^Q^177^K^178^ motif of CSB is dispensable for restart of stalled forks in both BRCA2-proficient and BRCA2-deficient cells

We have previously reported that CSB promotes restart of stalled forks (17). To investigate whether CSB relies on the R^176^Q^177^K^178^ motif to restart stalled forks, U2OS CSB-KO cells transfected with the vector alone, Myc-CSB or Myc-CSB-R^176^Q^177^K^178^-AAA were first labeled with IdU for 20 min, treated with 4 mM HU for 4 h and then labeled with CldU for 40 min. Myc-CSB-R^176^Q^177^K^178^-AAA behaved indistinguishably from Myc-CSB in reducing the number of stalled forks in U2OS CSB-KO cells (Supplementary Figure S8A). The R^176^Q^177^K^178^-AAA mutations also did not affect the ability of Myc-CSB to reduce the number of stalled forks in BRCA2-depleted U2OS CSB-KO cells (Supplementary Figure S8B). These results altogether suggest that this RQK motif is dispensable for CSB to mediate restart of stalled forks in both BRCA2-proficient and BRCA2-deficient cells.

### CSB competes with SMARCAL1 for RPA32 at stalled forks

**Figure 5.**
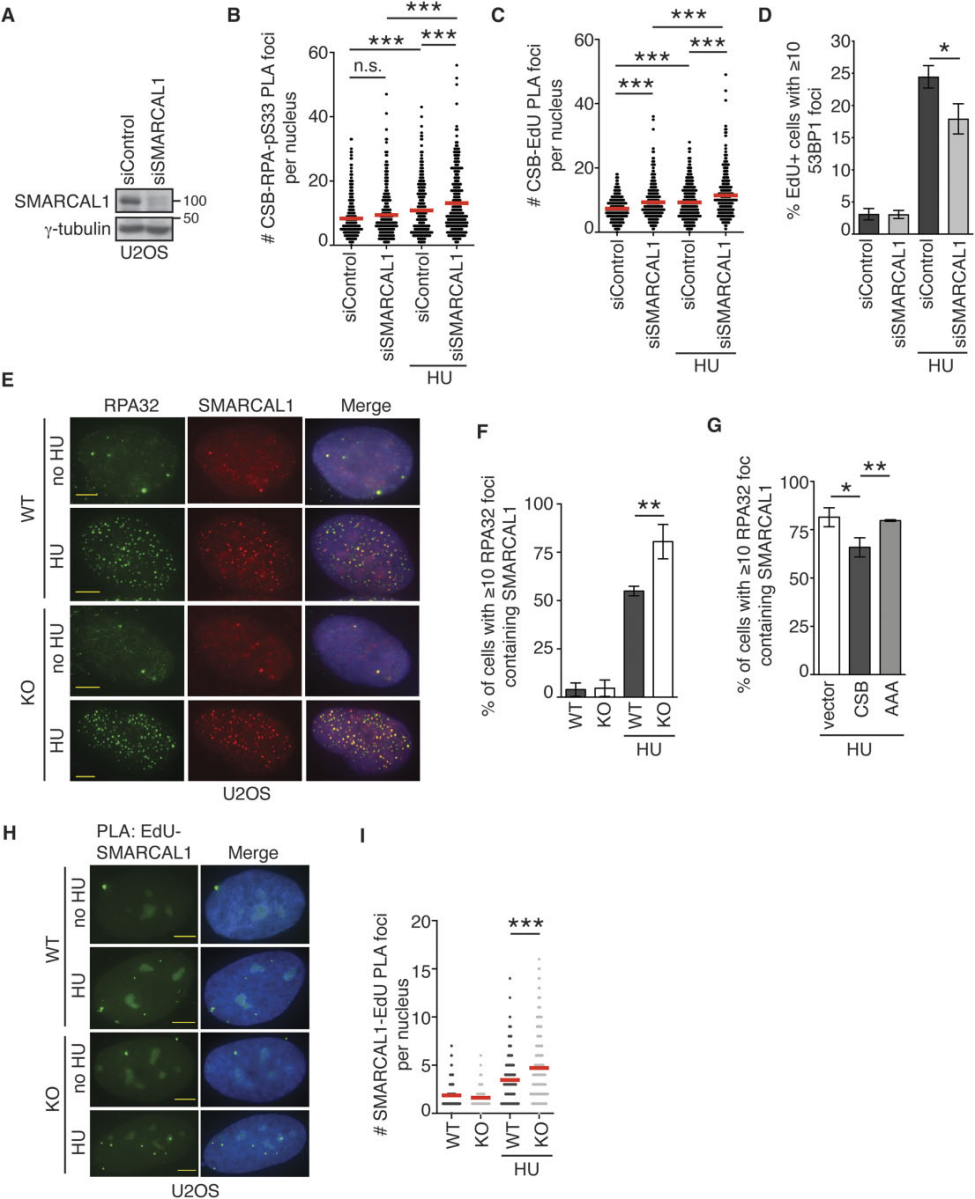
CSB competes with SMARCAL1 for RPA at stalled forks. (**A**) Western analysis of U2OS cells transfected with siControl or siSMARCAL1. Immunoblotting was done with anti-SMARCAL1 and anti-γ-tubulin antibodies. (**B**) Quantification of PLA between CSB and RPA-pS33 in siControl- or siSMARCAL1-transfected U2OS cells in the presence or the absence of HU. The respective number of cells analyzed for siControl (–HU), siSMARCAL1 (–HU), siControl (+HU), and siSMARCAL1 (+HU) were 313, 318, 355 and 308. Data from single experiments are represented as scatter plot graphs with the mean indicated in this and 5C panels. The *P*-value was determined using a non-parametric Mann–Whitney rank-sum *t*-test in this and 5C panels. ***P< 0.001. (**C**) Quantification of PLA between CSB and EdU in siControl- or siSMARCAL1-transfected U2OS cells in the presence or the absence of HU. For HU treatment, cells were fixed 4 h post treatment with 4 mM HU. The respective number of cells analyzed for siControl (–HU), siSMARCAL1 (–HU), siControl (+HU), and siSMARCAL1 (+HU) were 299, 336, 350 and 303. ***P< 0.001. (**D**) Quantification of the percentage of EdU+ cells exhibiting ≥ 10 53BP1 foci. U2OS cells transfected with indicated siRNAs were pulse-labeled with EdU for 10 min and then treated with or without 4 mM HU for 4 h. A total of 503–539 cells per condition were scored in a blind manner. Standard deviations from three independent experiments were shown. *P< 0.05. (**E**) Representative images of U2OS CSB-WT and CSB-KO cells that were treated with or without HU. Immunostaining was done with an anti-RPA32 antibody (green) in conjunction with an anti-SMARCAL1 (red) antibody. (**F**) Quantification of the percentage of U2OS CSB-WT and CSB-KO cells exhibiting ≥ 10 colocalization foci of RPA32 and SMARCAL1 from (E). A total of 500–558 cells were scored per condition in a blind manner. SDs from three independent experiments are indicated in this and (G) panels. **P< 0.01. (**G**) Quantification of the percentage of vector-, Myc-CSB-, or Myc-CSB-R^176^Q^177^K^178^-AAA (AAA)-expressing U2OS CSB-KO cells exhibiting ≥10 colocalization foci of RPA32 and SMARCAL1. A total of 501–528 cells were scored per condition in a blind manner. *P< 0.05; **P< 0.01. (**H**) Representative images of PLA foci formation between SMARCAL1 and EdU in U2OS CSB-WT and CSB-KO cells that were treated with or without HU. (**I**) Quantification of PLA between SMARCAL1 and EdU in U2OS WT and CSB-KO cells treated with or without HU. The respective number of cells analyzed for WT (–HU), KO (–HU), WT (+HU), and KO (+HU) were 297, 287, 296 and 308. Data from single experiments are represented as scatter plot graphs with the mean indicated. The *P*-value was determined using a non-parametric Mann–Whitney rank-sum *t*-test. ***P< 0.001.

It has been reported that SMARCAL1 is recruited by RPA32 to stalled replication forks (14). We have shown that efficient association of CSB with stalled forks is dependent upon its interaction with RPA32, prompting us to ask if CSB and SMARCAL1 compete with each other to bind RPA32 at stalled forks. To address this question, we first knocked down SMARCAL1 in U2OS cells (Figure 5A), followed by measurement of PLA foci formation between CSB and RPA32-pS33. Depletion of SMARCAL1 stimulated the formation of CSB-RPA32-pS33 PLA foci in U2OS cells in response to treatment with HU (Figure 5B), suggesting that SMARCAL1 inhibits the CSB-RPA32 interaction at stalled forks. In agreement with the notion that the CSB-RPA32 interaction mediates CSB’s recruitment to stalled forks, depletion of SMARCAL1 also increased the number of CSB-EdU PLA foci in HU-treated U2OS cells (Figure 5C). Loss of SMARCAL1 has been reported to lead to persistently stalled replication forks (62,63), which can be processed to generate DSBs. However, under our experimental conditions, we did not detect any increase in the foci formation of 53BP1, a marker for DSBs, in EdU+ SMARCAL1-depleted U2OS cells following treatment with HU for 4 h (Figure 5D). Instead, depletion of SMARCAL1 led to a mild decrease in the number of EdU+ cells exhibiting 53BP1 foci formation following treatment with HU (Figure 5D). These results suggest that the increased recruitment of CSB to stalled forks in SMARCAL1-depleted cells is unlikely to be mediated by DSBs. Interestingly, we also observed that depletion of SMARCAL1 enhanced the number of CSB-EdU PLA foci in untreated U2OS cells, indicating that SMARCAL1 may inhibit CSB’s association with ongoing forks.

To investigate whether CSB regulates the SMARCAL1–RPA32 interaction at stalled forks, we measured HU-induced colocalization of SMARCAL1 with RPA32 in both U2OS CSB-WT and CSB-KO cells since SMARCAL1 is recruited by RPA32 to stalled replication forks, forming damage-induced foci that colocalize with RPA32 (14). In agreement with a previous finding (14), SMARCAL1 formed HU-induced damage foci that colocalized with RPA32 in U2OS CSB-WT cells (Figure 5E and F). This colocalization was further increased in U2OS CSB-KO cells (Figure 5E and F). To further substantiate this finding, we pulse-labeled both U2OS CSB-WT and CSB-KO cells with EdU in the presence or absence of HU and quantified the formation of PLA foci between SMARCAL1 and EdU. Loss of CSB increased the number of HU-induced SMARCAL1-EdU PLA foci (Figure 5H and I). These results altogether suggest that CSB inhibits RPA32-dependent recruitment of SMARCAL1 to stalled forks.

To further investigate whether CSB relies on its RPA32-interacting R^176^Q^177^K^178^ to regulate the SMARCAL1-RPA32 interaction at stalled forks, we quantified HU-induced colocalization of SMARCAL1 with RPA32 in U2OS CSB-KO cells expressing the vector alone, Myc-CSB or Myc-CSB carrying the R^176^Q^177^K^178^-AAA mutations. While overexpression of Myc-CSB into CSB-KO cells reduced HU-induced colocalization of SMARCAL1 and RPA32, this reduction was not observed in CSB-KO cells expressing Myc-CSB-R^176^Q^177^K^178^-AAA (Figure 5G), suggesting that binding of CSB to RPA32 interferes with the SMARCAL1-RPA32 interaction at stalled forks. Taken together, these results suggest that CSB and SMARCAL1 compete with each other for RPA32 at stalled forks.

### CSB and SMARCAL1 function non-redundantly to restrain PRIMPOL-dependent fork progression in response to mild replication stress

**Figure 6.**
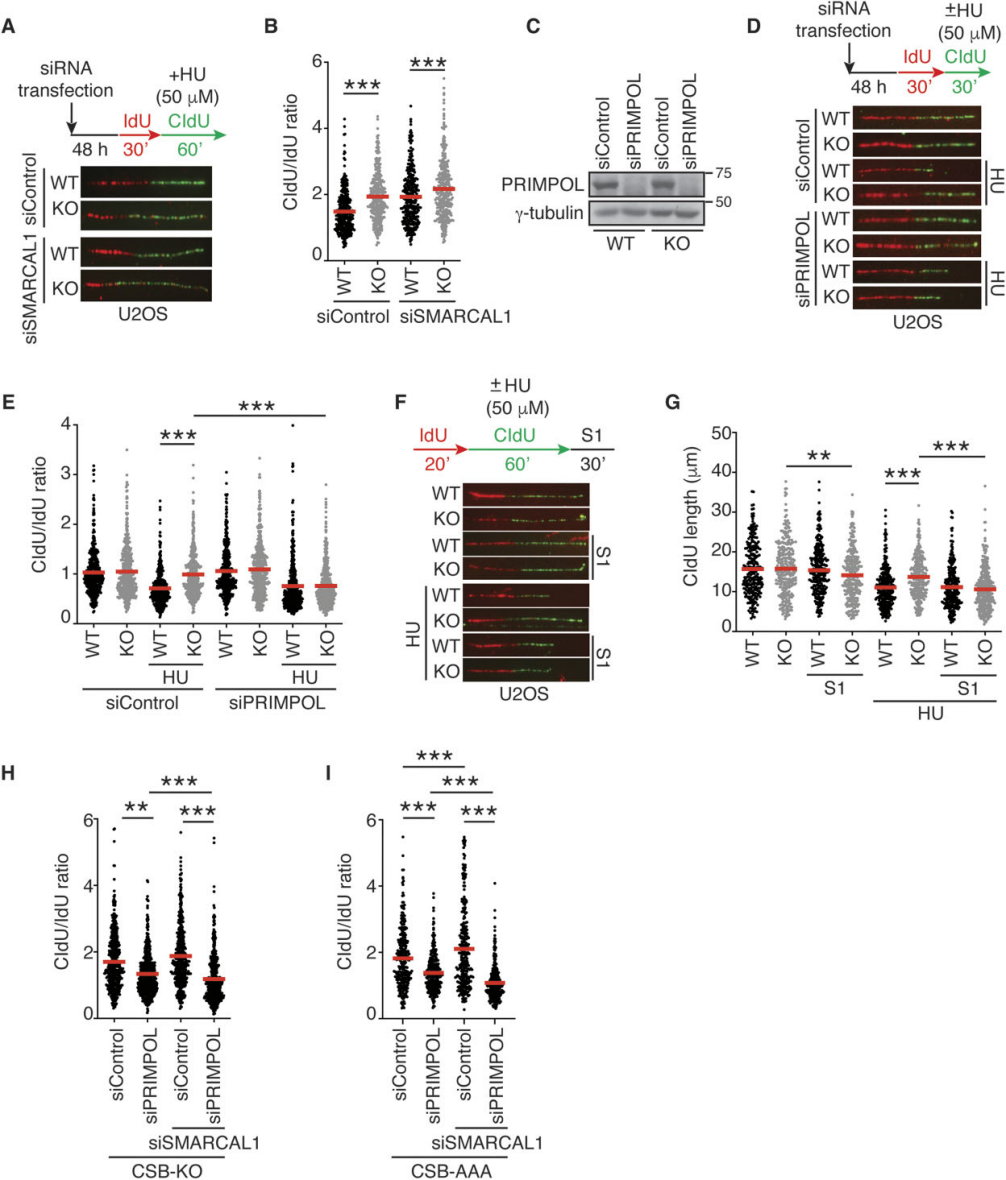
CSB and SMARCAL1 function non-redundantly at stalled forks to restrain fork progression. (**A**) Representative images of DNA fibers from U2OS WT and CSB knockout (KO) cells transfected with indicated siRNAs. Cells were first labeled with IdU (red) and then labeled with CldU (green) in the presence of 50 μM HU. (**B**) Quantification of the CldU/IdU ratio from U2OS CSB-WT and CSB-KO cells transfected with siControl or siSMARCAL1. A total of 306–313 fibers per condition were analyzed. Data from single experiments are represented as scatter plot graphs with the mean indicated in this and subsequent panels. The *P*-value was determined using a non-parametric Mann–Whitney rank-sum *t*-test in this, (E), (G–I) panels. ***P< 0.001. (**C**) Western analysis of U2OS CSB-WT and CSB-KO cells transfected with siControl or siPRIMPOL. Immunoblotting was performed with anti-PRIMPOL and anti-γ-tubulin antibodies. ***P< 0.001. (**D**) Representative images of DNA fibers from U2OS WT and CSB knockout (KO) cells transfected with indicated siRNAs. Cells were first labeled with IdU (red) and then labeled with CldU (green) in the presence of 50 μM HU. (**E**) Quantification of the CldU/IdU ratio from U2OS CSB-WT and CSB-KO cells transfected with siControl or siPRIMPOL. A total of 403–478 fibers per condition were analyzed. ***P< 0.001. (**F**) Representative images of DNA fibers from U2OS WT and CSB knockout (KO) cells. Following the second labeling with CldU (green) in the presence or absence of 50 μM HU, cells were treated with S1 nuclease for 30 min. (**G**) Quantification of the CldU fiber length from U2OS CSB-WT and CSB-KO cells following treatment with S1 nuclease. A total of 255–345 fibers per condition were analyzed. **P< 0.01; ***P< 0.001. (**H**) Quantification of the CldU/IdU ratio from U2OS CSB-KO cells transfected with siControl, siPRIMPOL, siSMARCAL1, or a combination of siPRIMPOL and siSMARCAL1. A total of 455–494 fibers per condition were analyzed. **P< 0.01; ***P< 0.001. (**I**) Quantification of the CldU/IdU ratio. U2OS CSB-KO cells expressing Myc-CSB-R^176^Q^177^K^178^-AAA (AAA) were transfected with siControl, siPRIMPOL, siSMARCAL1, or a combination of siPRIMPOL and siSMARCAL1. A total of 300–303 fibers per condition were analyzed. ***P< 0.001.

Both CSB and SMARCAL1 have been implicated in catalyzing fork reversal to slow down fork progression upon mild replication stress (15,17). To further investigate the genetic relationship between CSB and SMARCAL1 at stalled forks, we asked whether CSB and SMARCAL1 function non-epistatically to restrain fork progression upon replication stress since we have shown that they compete each other at stalled forks. We have previously reported that depletion of SMARCAL1 has little effect in fork progression in CSB-KO cells in the presence of 50 μM HU (17). In this previous study, cells were labeled first with IdU for 30 min and then with CldU for 30 min in the presence of 50 μM HU. Fork progression was measured by the ratio of CldU/IdU, which has a maximum number of 1. We questioned whether this limit might have masked a possible non-redundant effect of CSB and SMARCAL1 on fork progression. To address this issue, we re-examined fork progression in SMARCAL1-depleted U2OS CSB-KO cells using a modified fork progression assay. In this modified assay, we labeled cells with IdU for 30 min and then with CldU for 60 min in the presence of 50 μM HU, allowing a wider range in the ratio of CldU/IdU. Using this modified assay, depletion of SMARCAL1 led to a further increase in the ratio of CldU/IdU in U2OS CSB-KO cells compared to U2OS CSB-WT cells in response to treatment with 50 μM HU (Figure 6A and B). These results suggest that CSB and SMARCAL1 act in parallel to restrain fork progression, which is in agreement with our previous finding that CSB and SMARCAL1 function non-epistatically to resolve replication stress at ALT telomeres (32).

It has been reported that PRIMPOL-dependent fork repriming mediates unrestrained fork progression in the absence of SMARCAL1 in response to replication stress (64). To investigate whether PRIMPOL mediates unrestrained fork progression in CSB-KO cells, we knocked down PRIMPOL in both U2OS WT and CSB-KO cells (Figure 6C). DNA fiber analysis revealed that depletion of PRIMPOL abrogated the restoration of the ratio of CldU/IdU in CSB-KO cells in the presence of 50 μM HU (Figure 6D and E), in agreement with our previous finding that PRIMPOL mediates unrestrained fork progression in CSB-KO cells in response to a low dose of camptothecin (CPT) (36). PRIMPOL-mediated fork repriming is associated with accumulation of ssDNA gaps (64–66). We found that restored DNA fiber length in CSB-KO cells in the presence of 50 μM HU was sensitive to treatment with S1 nuclease (Figure 6F and G). To further substantiate whether PRIMPOL is responsible for unrestrained fork progression in cells lacking both SMARCAL1 and CSB, we co-depleted PRIMPOL and SMARCAL1 in CSB-KO cells. We observed that the synthetic increase in the ratio of CldU/IdU in SMARCAL1-depleted CSB-KO cells was sensitive to depletion of PRIMPOL (Figure 6H). Interestingly, the ratio of CldU/IdU in CSB-KO cells depleted for both SMARCAL1 and PRIMPOL was further reduced compared to CSB-KO cells depleted for PRIMPOL alone (Figure 6H). Taken together, these results suggest that CSB and SMARCAL1 act non-redundantly to restrain PRIMPOL-mediated fork repriming.

We have shown that CSB binds RPA32 through its R^176^Q^177^K^178^ motif and competes with SMARCAL1 for RPA32. To investigate whether CSB relies on its R^176^Q^177^K^178^ motif to function non-redundantly with SMARCAL1 to restrain PRIMPOL-dependent fork progression, we knocked down SMARCAL1, PRIMPOL, or a combination of SMARCAL1 and PRIMPOL in U2OS CSB-KO cells expressing Myc-CSB-R^176^Q^177^K^178^-AAA. DNA fiber analysis revealed that the unrestrained fork progression in Myc-CSB-R^176^Q^177^K^178^-AAA-expressing CSB-KO cells was further exacerbated by depletion of SMARCAL1 as evidenced by the increased ratio of CldU/IdU (Figure 6I). This exacerbation was sensitive to depletion of PRIMPOL (Figure 6I). Similar to CSB-KO cells, depletion of both SMARCAL1 and PRIMPOL led to a further reduction in the ratio of CldU/IdU in U2OS CSB-KO cells expressing Myc-CSB-R^176^Q^177^K^178^-AAA (Figure 6I). These results suggest that the R^176^Q^177^K^178^ motif of CSB mediates its synergistic interaction with SMARCAL1 to restrain fork progression upon replication stress.

### Unlike CSB, SMARCAL1 inhibits rather than promotes fork restart and genomic stability in BRCA2-deficient cells

**Figure 7.**
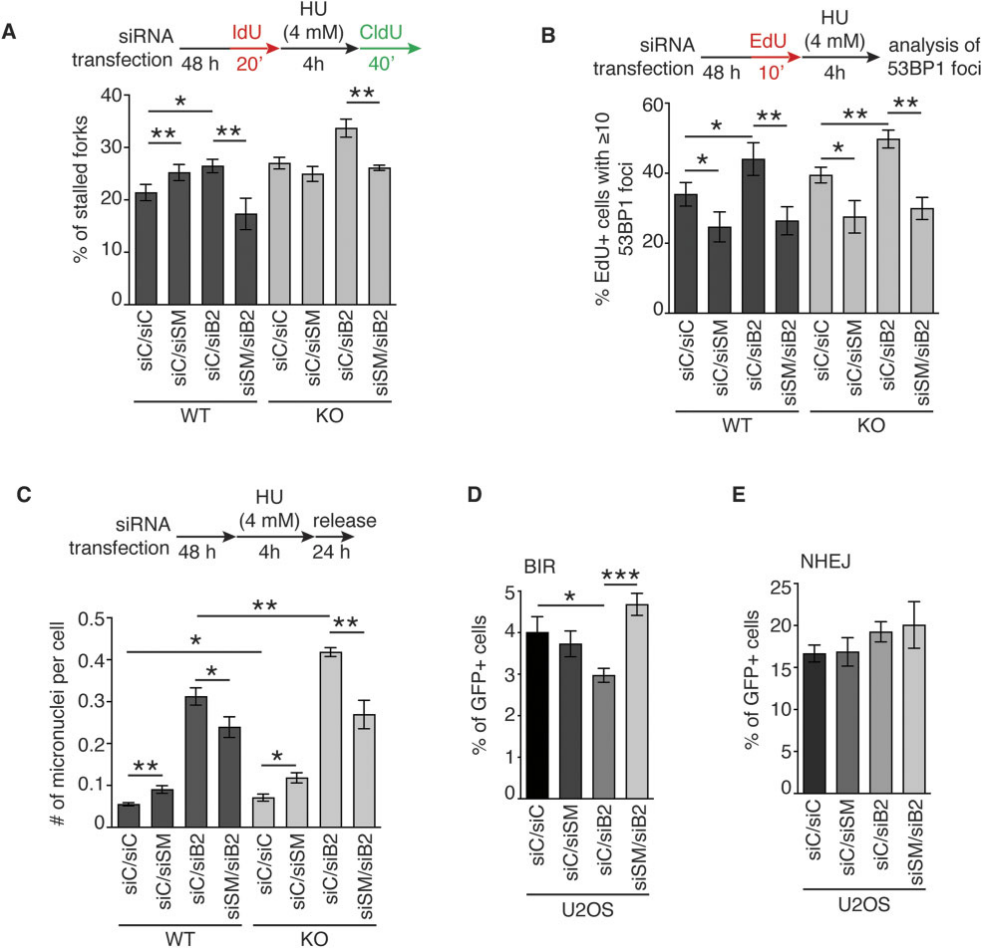
CSB and SMARCAL1 differentially regulate the restart of stalled forks in BRCA2-deficient cells. (**A**) Quantification of the percentage of stalled forks from U2OS CSB-WT and CSB-KO cells transfected with indicated siRNAs. A total of 340–471 fibres per condition were scored in a blind manner. SDs from three independent experiments are shown. *P< 0.05; **P< 0.01. (**B**) Quantification of the percentage of cells exhibiting ≥10 53BP1 foci. U2OS WT and CSB-KO cells transfected with indicated siRNAs were pulse-labeled with EdU for 10 min prior to treatment with 4 mM HU for 4 h. A total of 504–531 cells per condition were scored in a blind manner. SDs from three independent experiments were shown. *P< 0.05; **P< 0.01. (**C**) Quantification of the average number of micronuclei per cell. U2OS WT and CSB-KO cells transfected with indicated siRNAs were treated with 4 mM HU and then released for 24 h. A total of 1005–1043 cells per condition were scored in a blind manner. SDs from three independent experiments were shown. *P< 0.05; **P< 0.01. (**D**) Quantification of the percentage of cells with the restoration of GFP expression following BIR-mediated repair of I-SceI-induced DSBs. SDs from three independent experiments are shown. *P< 0.05; ***P< 0.001. (**E**) Quantification of the percentage of cells with the restoration of GFP expression following NHEJ-mediated repair of I-SceI-induced DSBs. SDs from three independent experiments are shown.

We have previously reported that loss of CSB impairs restart of stalled forks and that this impairment is further exacerbated in BRCA2-deficient cells (17), indicative of a synthetic sick interaction between CSB and BRCA2 in regulating the restart of stalled forks. It has been reported that SMARCAL1 promotes restart of stalled forks (14), however little is known about its role in fork restart in BRCA2-deficient cells. Thus, we asked whether CSB and SMARCAL1 function epistatically to promote restart of stalled forks in BRCA2-deficient cells. To address this question, we transfected U2OS CSB-WT and CSB-KO cells with either siControl, siSMARCAL1, siBRCA2, or a combination of siSMARCAL1 and siBRCA2. These cells were first labeled with IdU for 20 min, treated with 4 mM HU for 4 h and then labeled with CldU for 40 min. Analysis of DNA fiber assays revealed that in CSB-WT cells, knockdown of SMARCAL1 led to a mild but significant increase in the number of stalled forks (Figure 7A), in agreement with a previous report that SMARCAL1 promotes fork restart (14). Depletion of BRCA2 in CSB-WT cells also increased the number of stalled forks (Figure 7A), in agreement with our previous finding (17). Interestingly, co-depletion of SMARCAL1 and BRCA2 suppressed the accumulation of stalled forks in CSB-WT cells (Figure 7A), indicative of a synthetic rescue interaction between SMARCA1 and BRCA2 in promoting fork restart. On the other hand, we observed a synthetic sick interaction between CSB and BRCA2 in promoting fork restart since loss of CSB and depletion of BRCA2 led to a synergistic increase in the number of stalled forks (Figure 7A), in agreement with our previous finding (17). Depletion of SMARCAL1 eliminated the increase in the number of stalled forks induced by depletion of BRCA2 in CSB-KO cells (Figure 7A), suggesting that loss of SMARCAL1 suppresses accumulation of stalled forks in the absence of BRCA2 in a manner independent of CSB. Taken together, these results suggest that CSB and SMARCAL1 are engaged in distinct genetic interactions with BRCA2 in regulating fork restart. These results further suggest that SMARCAL1 inhibits rather than promotes fork restart under the pathological condition lacking functional BRCA2.

Stalled forks can collapse, generating DNA double strand breaks (DSBs) and driving genomic instability. To investigate whether HU-induced stalled forks are associated with accumulation of DSBs, we transfected both U2OS CSB-WT or CSB-KO cells with siControl, siSMARCAL1, siBRCA2 or a combination of siSMARCAL1 and siBRCA2. These cells were then pulse-labeled with EdU, treated with 4 mM HU for four hours prior to fixation for immunofluorescence analysis of 53BP1 foci formation. We found that loss of CSB had little impact on the number of EdU+ cells exhibiting ≥10 53BP1 foci in U2OS cells following treatment with HU (Figure 7B). On the other hand, depletion of SMARCAL1 led to a mild decline in the number of EdU+ cells exhibiting ≥ 10 53BP1 foci whereas depletion of BRCA2 increased the number of EdU+ cells exhibiting ≥10 53BP1 foci in U2OS cells irrespectively of the status of CSB (Figure 7B). These results suggest that HU-induced stalled forks are likely to be processed into DSBs in the absence of BRCA2 but not in the absence of CSB alone or SMARCAL1 alone during the 4-h time period of HU treatment. Depletion of SMARCAL1 led to a pronounced reduction in the number of EdU+ cells exhibiting ≥10 53BP1 foci in BRCA2-depleted U2OS cells irrespectively of the status of CSB following the treatment with HU (Figure 7B), suggesting that SMARCAL1 promotes accumulation of DSBs upon replication stress in the absence of BRCA2. In agreement with this notion, depletion of SMARCAL1 reduced the formation of micronuclei in BRCA2-depleted U2OS cells irrespective of the status of CSB following treatment with HU (Figure 7C). In contrast, depletion of CSB further exacerbated the formation of micronuclei in BRCA2-depleted cells following treatment with HU (Figure 7C). Taken together, these results suggest that unlike CSB, SMARCAL1 promotes genomic instability in BRCA2-deficient cells upon replication stress.

### SMARCAL1 inhibits BIR in BRCA2-deficient cells

In the absence of BRCA2, reversed forks are known to be processed into broken forks (22–24), which can be repaired by break-induced replication (BIR) to restart DNA synthesis (17). Our finding that SMARCAL1 inhibits restart of stalled forks in BRCA2-deficient cells prompted us to ask whether this inhibition is associated with a change in the activity of BIR in BRCA2-deficient cells. To address this question, we turned to a previously-described BIR reporter plasmid (pBIR-GFP) (43), in which restoration of GFP expression requires BIR repair of an I-SceI-induced DSB in the GFP gene. We measured BIR-dependent restoration of GFP expression in U2OS cells transfected with siControl, siSMARCAL1, siBRCA2 or a combination of siSMARCAL1 and siBRCA2. It has been reported that inactivation of BRCA2 reduces BIR efficiency (67). In agreement with this previous finding, we observed that depletion of BRCA2 impaired BIR-dependent restoration of GFP expression (Figure 7D). This impairment was completely suppressed by depletion of SMARCAL1. Depletion of SMARCAL1 had little impact on BIR-dependent restoration of GFP expression in U2OS cells that were transfected with siControl (Figure 7D). These results suggest that SMARCAL1 inhibits BIR under the pathological condition lacking functional BRCA2.

SMARCAL1 has been reported to promote NHEJ (68). To investigate whether SMARCAL1 promotes NHEJ to inhibit BIR, we used a previously-described GFP-based NHEJ reporter plasmid (pEGFP-Pem1-Ad2) (30,42), in which restoration of GFP expression requires NHEJ repair of an I-SceI-induced DSB in the GFP gene. We found that depletion of SMARCAL1 had no effect in NHEJ-dependent restoration of GFP expression in U2OS cells with or without depletion of BRCA2 (Figure 7E), which was not in agreement with the previous report (68). This discrepancy is likely due to the difference in the experimental conditions, e.g. SMARCAL1 knockout in DT40 and B cells in the previous work versus SMARCAL1 knockdown in U2OS cells here. Nevertheless, these results suggest that it is unlikely that SMARCAL1 promotes NHEJ-mediated repair of I-SceI-induced DSBs.

### Loss of CSB resensitizes BRCA2-deficient cells that have acquired chemoresistance through loss of SMARCAL1

**Figure 8.**
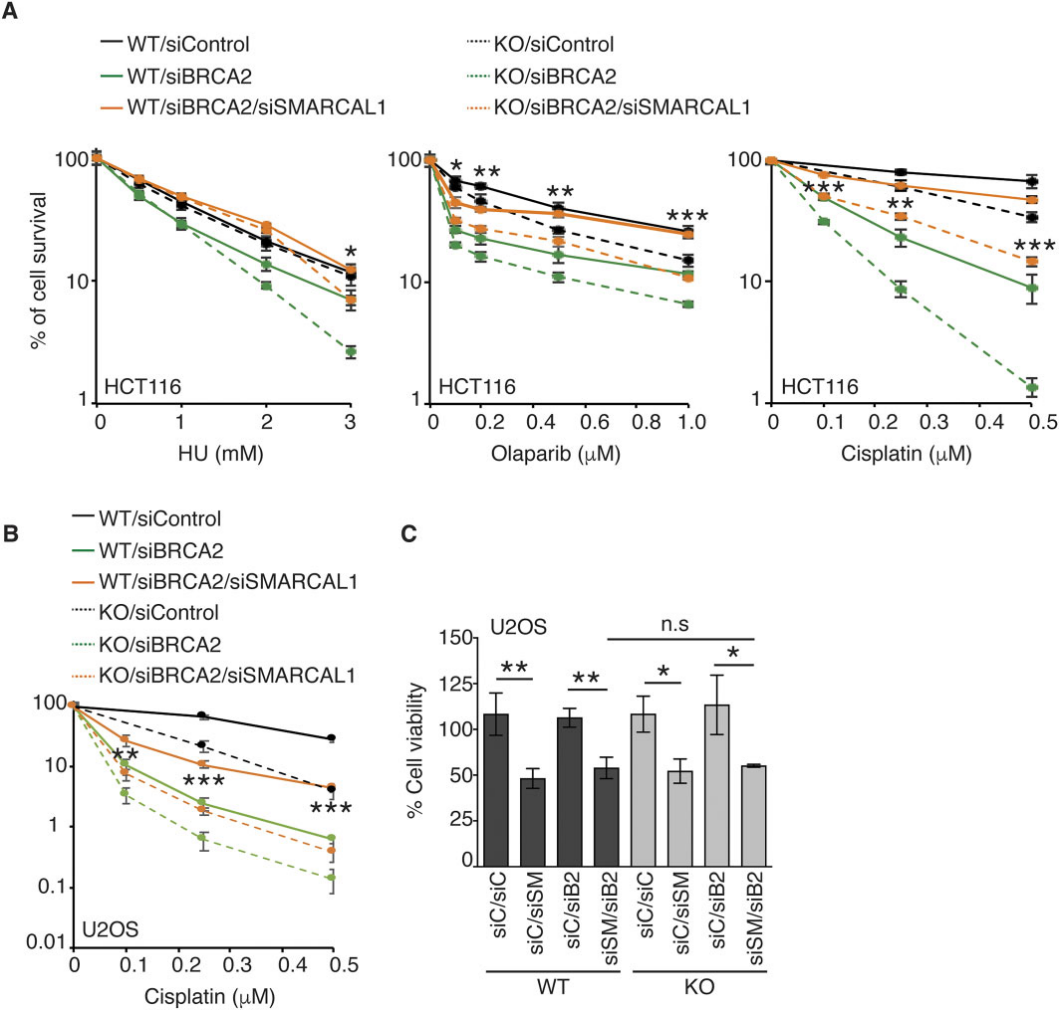
CSB and SMARCAL1 differentially regulate the chemoresponse in BRCA2-deficient cells. (**A**) Clonogenic survival assays of HCT116 cells as indicated to HU, olaparib, and cisplatin as indicated. SDs from three independent experiments are indicated. *P* values for comparison between CSB-WT/siBRCA2/siSMARCAL1 and CSB-KO/siBRCA2/siSMARCAL1 are indicated. *P< 0.05; **P< 0.01; ***P< 0.001. (**B**) Cisplatin clonogenic survival assays of U2OS WT and CSB-KO cells transfected with indicated siRNAs. SDs from three independent experiments are indicated. *P* values for comparsion between CSB-WT/siBRCA2/siSMARCAL1 and CSB-KO/siBRCA2/siSMARCAL1 are indicated. **P< 0.01; ***P< 0.001. (**C**) Cell viability assays of U2OS WT and CSB-KO cells transfected with indicated siRNAs. SDs from three independent experiments are indicated. *P< 0.05; **P< 0.01; n.s., not significant.

Both CSB and SMARCAL1 have been implicated in regulating the chemoresponse in BRCA2-deficient cells. While CSB exacerbates chemosensitivity in BRCA2-deficient cells (17), loss of SMARCAL1 has been reported to confer chemoresistance in BRCA2-deficient cells (19,69). To investigate whether CSB could resensitize SMARCAL1-depleted BRCA2-deficient cells, we knocked down SMARCAL1 in both HCT116 CSB-WT and CSB-KO cells that were also depleted with BRCA2. Analysis of clonogenic survival assays revealed that depletion of SMARCAL1 conferred resistance to HU, olaparib, or cisplatin in BRCA2-depleted HCT116 CSB-WT cells (Figure 8A), in agreement with a previous finding (19). This chemoresistance was largely abrogated by loss of CSB (Figure 8A), suggesting that inhibiting CSB can restore chemosensitivity in SMARCAL1-depleted BRCA2-deficient HCT116 cells. The ability of loss of CSB to restore chemosensitivity was also observed in another cell line U2OS depleted of both SMARCAL1 and BRCA2 (Figure 8B). This restored chemosensitivity is unlikely to be due to a change in the ability of cells to proliferate since loss of CSB had little impact on the cell viability in U2OS depleted of both SMARCAL1 and BRCA2 under unperturbed conditions (Figure 8C). We observed that CSB-KO cells depleted for SMARCAL1 in combination with BRCA2 were still more resistant to HU, olaparib, or cisplatin than CSB-KO cells depleted for BRCA2 alone (Figure 8A and B), suggesting that loss of SMARCAL1 counteracts the effect of loss of BRCA2 but not loss of CSB on the chemoresponse.

## Discussion

DNA translocases that are members of the SNF2 helicase family have been implicated in fork reversal, including but not limited to SMARCAL1, ZRANB3, HLTF and CSB (15,17,47,70). SMARCAL1 interacts with RPA and is recruited by RPA32 to stalled forks (13–15). ZRANB3 is recruited to stalled forks through an interaction of PCNA instead of RPA (71,72). However, how CSB is recruited to stalled forks is poorly understood. The work presented here has uncovered that CSB interacts with RPA and that this interaction is likely of low abundance and/or transient. Our finding suggests that CSB is recruited to stalled forks through at least in part a direct interaction between RPA32 and an RPA32-interacting motif within the N-terminal region of CSB. In addition, our finding suggests that CSB recruitment to stalled forks is unlikely mediated by DSBs arising from collapse of stalled forks.

We have previously reported that CDK phosphorylates CSB on T1031 and that this phosphorylation is dispensable for fork restart but necessary to promote MRE11-mediated fork degradation in BRCA2-deficient cells (17). Our finding presented here suggests that the R^176^Q^177^K^178^ motif of CSB is epistatic to CSB phosphorylation on T1031 to recruit CSB to stalled forks as well as to promote fork slowing and MRE11-dependent fork degradation in BRCA2-deficient cells. Both fork slowing and fork degradation in BRCA2-deficient cells are indirect readouts of fork reversal activity *in vivo*. We have previously reported that CSB possesses an intrinsic fork reversal activity, which is likely to be highly regulated *in vivo* (17). Our finding that RPA32 binding-defective CSB mutant (R^176^Q^177^K^178^-AAA) fails to promote not only fork slowing but also fork degradation in BRCA2-deficient cells suggests that the CSB-RPA interaction is likely engaged in the regulation of CSB’s ability to promote fork reversal. We have shown that the CSB-R^176^Q^177^K^178^-AAA mutant is fully competent in promoting the restart of stalled forks, similar to the CSB-T1031A mutant (17), supporting our previously-published notion that CSB’s role in fork reversal is mechanistically separable from CSB’s role in fork restart. It has been reported that RPA directs SMARCAL1 to selectively regress stalled forks caused by blockage to the leading strand polymerase (55). Conceivably, RPA could play a similar role in directing CSB to selectively regress stalled forks, which would require future investigation.

RPA32 contains a C-terminal domain that adopts a winged-helix-turn-helix fold known to be engaged in protein-protein interactions (58). Protein interactions of RPA32C with several DNA replication and repair proteins such as SMARCAL1, RAD52, UNG2, and XPA have been characterized structurally (58,60,73). The AlphaFold2 modeling presented here and its comparison to experimental structures of SMARCAL1 and UNG2 bound to RPA32 suggests that a highly conserved region in the N-terminal region of CSB, where the R^176^Q^177^K^178^ motif is located, interacts with RPA32C, likely in a manner similar to these factors. The variations observed in the CSB-RPA32 AlphaFold predictions also reinforce the view that a significant plasticity is allowed in RPA32C binding.

**Figure 9.**
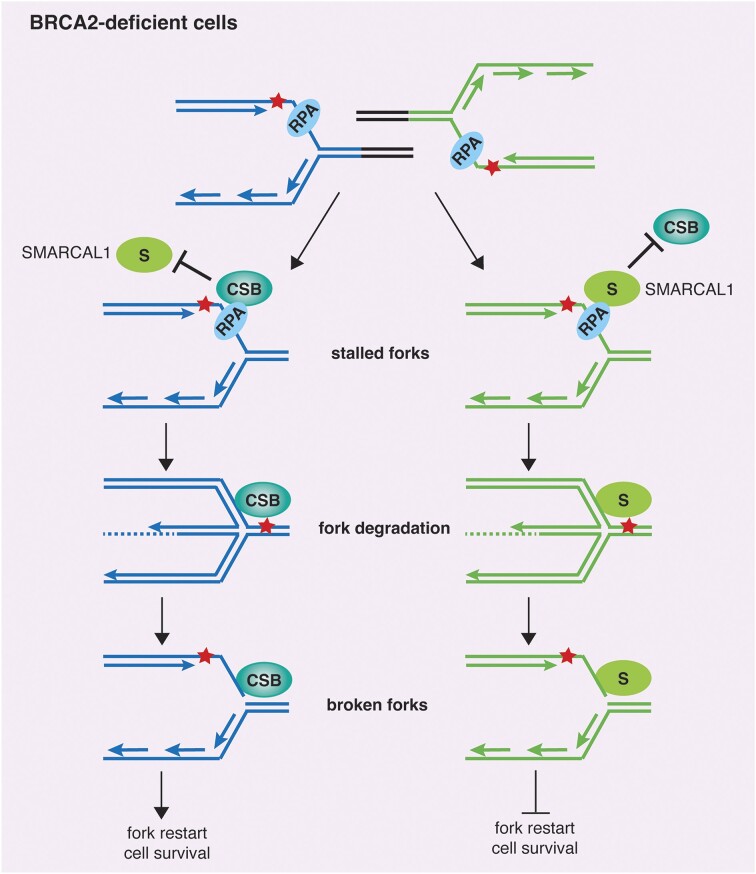
Model for differential control of the fate of stalled forks by CSB and SMARCAL1 under the pathological condition lacking BRCA2. CSB and SMARCAL1 compete for RPA, which interacts and directs them to different types of stalled forks as depicted by diagrams of stalled forks in different colors. See the text for additional details.

It has been well documented that PRIMPOL-mediated fork repriming generates ssDNA gaps (64–66), which if not repaired properly, can threaten genomic stability (74). Our finding suggests that CSB and SMARCAL1 act synergistically to restrain PRIMPOL-dependent fork repriming, thereby preventing excessive formation of ssDNA gaps in response to replication stress. Our finding that CSB competes with SMARCAL1 for RPA32 at stalled forks suggests that CSB and SMARCAL1 are unlikely to be recruited to the same stalled forks. We have shown that while CSB is engaged in a synthetic sick interaction with BRCA2 in restarting stalled forks, SMARCAL1 is engaged in a synthetic rescue interaction with BRCA2 in restarting stalled forks. These findings altogether lead us to propose that RPA32 directs initial recruitment of CSB and SMARCAL1 to distinct types of stalled forks to control their fates, particularly under the pathological condition lacking functional BRCA2 (Figure 9). Once recruited to stalled forks, both CSB and SMARCAL1 can remodel stalled forks into reversed forks, however, these reversed forks are known to be processed into broken forks in BRCA2-deficient cells. We have previously reported that CSB stimulates BIR-mediated repair and restart of broken forks in BRCA2-deficient cells (17). Thus, for CSB-associated broken forks, CSB stimulates their repair by BIR, which promotes chemoresistance in BRCA2-deficient cells. On the other hand, our work presented here suggests that SMARCAL1 inhibits restart of stalled forks in BRCA2-deficient cells, likely by suppressing BIR-mediated repair of collapsed forks.

How does SMARCAL1 inhibit BIR in BRCA2-deficient cells? It has been suggested that SMARCAL1 controls removal of histone acetylation marks and subsequent replacement by methylation marks during replication-coupled chromatin assembly, thereby ensuring the re-establishment of repressive chromatin (75). Conceivably, open chromatin resulting from loss of SMARCAL1 could favor homology-based repair such as BIR in BRCA2-deficient cells. SMARCAL1 has been reported to promote nonhomologous end joining (NHEJ) (68), raising the possibility that downregulation of NHEJ resulting from loss of SMARCAL1 could channel DSBs towards repair by BIR in BRCA2-deficient cells. While depletion of SMARCAL1 does not affect NHEJ-mediated repair of I-SceI-induced DSBs (Figure 7E), depletion of SMARCAL1 decreases HU-induced foci formation of 53BP1, a NHEJ-promoting factor (76), in S phase as marked by EdU (Figures 5D and 7B). The latter supports the notion that SMARCAL1 promotes NHEJ to inhibit BIR-mediated restart of stalled forks in BRCA2-deficient cells. Future studies would be needed to investigate the nature of SMARCAL1 mediated BIR inhibition in BRCA2-deficient cells.

Chemoresistance is a major challenge in cancer treatment. Chemoresistance in BRCA2-deficient cells can arise from loss of SMARCAL1, which has been attributed to restoration of fork stability (19). Our finding suggests that loss of inhibition of restart of stalled forks could represent an alternative but non-mutually exclusive mechanism that contributes to chemoresistance induced by loss of SMARCAL1 in BRCA2-deficient cells. We have shown that loss of CSB restores chemosensitivity in cells depleted with both SMARCAL1 and BRCA2, which is likely due to a defect in CSB-mediated BIR repair of stalled forks (17). Our finding adds further evidence to growing lines of studies (35,77) suggesting that CSB is a promising target in targeted cancer therapy.

## Supplementary Material

gkae154_Supplemental_File

## Data Availability

All data used in this study are available within the article, Mendeley Data, Supplementary files, or available from the authors upon request. Alphafold Structure Dataset: DOI: Mendeley Data (https://doi.org/10.17632/psgm2fmrvt.1).
