# Supplementary material for: CSB and SMARCAL1 compete for RPA32 at stalled forks and differentially control the fate of stalled forks in BRCA2-deficient cells
Source: Nucleic Acids Res. 2024 Feb 28;52(9):5067–87. doi: 10.1093/nar/gkae154 (PMC11109976; doi:10.1093/nar/gkae154)
Supplement: gkae154_Supplemental_File [file gkae154_supplemental_file.pdf]

SUPPLEMENTARY FIGURE LEGENDS

Figure S1. MUS81 does not mediate HU-induced interaction of CSB with RPA32. (**A**) Coimmunoprecipitation with anti-CSB antibody in HCT116 cells. Immunoblotting was performed with anti-CSB and anti-RPA70 antibodies. (**B**) Western analysis of U2OS cells transfected with siControl or siMUS81. Immunoblotting was done with anti-MUS81 and anti-α-tubulin antibodies. The α-tubulin blot was used as a loading control. (**C**) Quantification of PLA foci formation between CSB and RPA32-pS4/S8 in U2OS cells transfected with indicated siRNAs. Cells were treated with or without 4 mM HU for 4 hours prior to fixation. A total of 295-343 cells per condition was scored in a blind manner. Data from single experiments are represented as scatter plot graphs with the mean indicated.

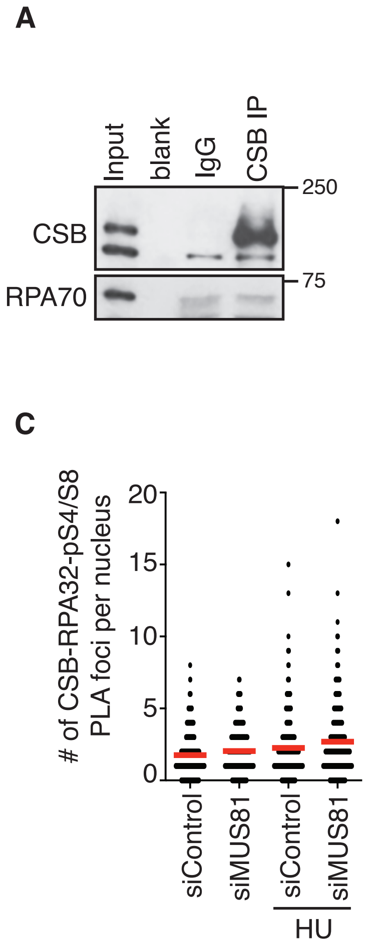

A
Input
blank
IgG
CSB IP
250
CSB
RPA70
75
B
siControl
siMUS81
75
MUS81
α-tubulin
50
C
# of CSB-RPA32-pS4/S8 PLA foci per nucleus
20
15
10
5
0
siControl
siMUS81
siControl
siMUS81
HU

Figure S2. The N-terminal region of CSB is engaged in an interaction with RPA. (**A**) Representative images of U2OS-265 CSB-KO cells expressing the vector alone, mCherry-LacR-CSB or mCherry-LacR-CSB carrying various deletions. Immunostaining was done with an anti-RPA32 antibody (green). Cell nuclei were stained with DAPI in blue in this and subsequent panels. Scale bars in this and subsequent panels: 5 μm. (**B**) Representative images of U2OS-265 CSB-KO cells expressing the vector alone, mCherry-LacR-CSB or mCherry-LacR-CSB carrying various deletions. Immunostaining was done with an anti-RPA70 antibody (green).

A

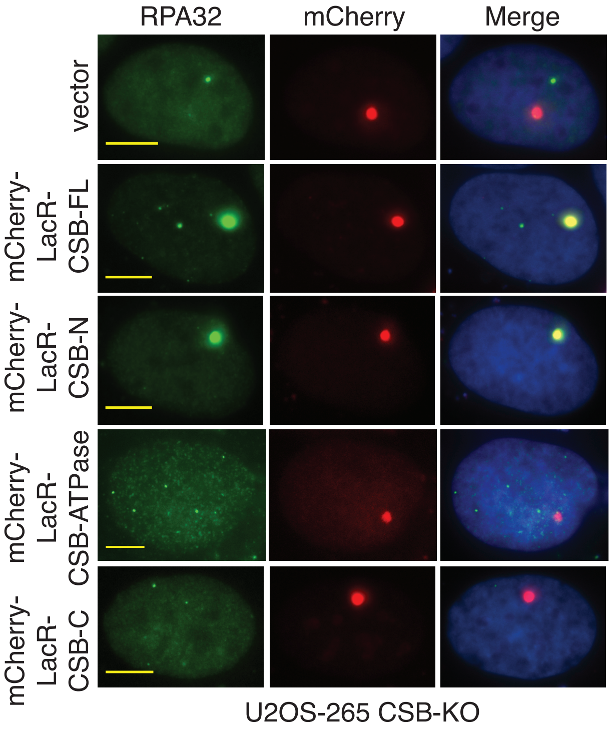
RPA32
mCherry
Merge
vector
mCherry-LacR-CSB-FL
mCherry-LacR-CSB-N
mCherry-LacR-CSB-ATPase
mCherry-LacR-CSB-C
U2OS-265 CSB-KO

B

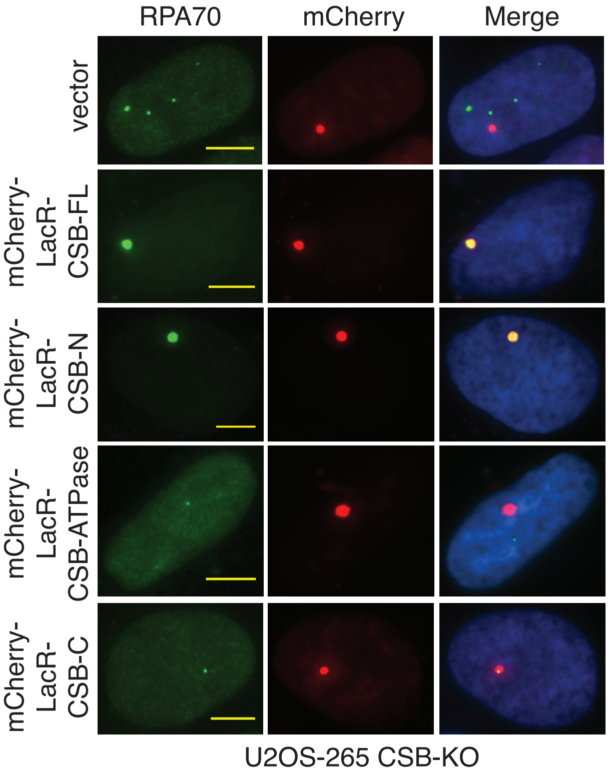
RPA70
mCherry
Merge
vector
mCherry-LacR-CSB-FL
mCherry-LacR-CSB-N
mCherry-LacR-CSB-ATPase
mCherry-LacR-CSB-C
U2OS-265 CSB-KO

Figure S3. The $R^{176}Q^{177}K^{178}$ motif but not the $R^{413}Q^{414}K^{415}$ motif mediates the interaction between CSB and RPA32. (**A**) The $R^{413}Q^{414}K^{415}$ motif, highlighted in yellow, diverges among CSB and its vertebrate orthologs as indicated by the sequence alignment. Accession numbers are: human ERCC-6, NP_001333369; dog ERCC-6 XP_534944.2; rat ERCC-6 NP_001100766.1; chicken ERCC-6, XP_421656.3; frog ERCC-6 NP_001361595.1; fish ERCC-6, XP_005815483.2 (**B**) Representative images of U2OS-265 CSB-KO cells expressing the vector alone, mCherry-LacR-CSB-N, mCherry-LacR-CSB-N carrying $R^{176}Q^{177}K^{178}$-AAA mutations (N-AAA) or mCherry-LacR-CSB-N carrying $R^{413}Q^{414}K^{415}$-AAA mutations (N-AAA2). Immunostaining was done with an anti-RPA32 antibody (green). Cell nuclei were stained with DAPI in blue. Scale bars: 5 μm. (**C**) Coimmunopreciptation with anti-Myc antibody in HEK293 cells transfected with mCherry-LacR-CSB carrying $R^{176}Q^{177}K^{178}$-AAA mutations (CSB-AAA) in conjunction with the vector alone or Myc-RPA70. Immunoblotting was performed with anti-Myc and anti-mCherry antibodies.

A

The $R^{413}Q^{414}K^{415}$ motif

```
                                                       AAA2
Human      391  AEADLSGD-GTDYELKPLPKG--GKRQKKVPVQE--IDDDFFPSSGE  433
Dog        394  VK--LTGE-DKDYELNPMLKR--WKRQKKVMVQE--NEDDFSPSSGE  433
Rat        367  TEAVLSSD-DISYELKPLRKG--QKYEKKVPVQE--IDDDFFPSSEE  408
Chicken    392  EQASSHAENDSDYELRNLSGK--GKYLVKRDSKDAGNDTDYFPSSEE  436
Frog       359  EEWVPESQ-DTEYELKPLIQR--TNGKRPR---ENGMDPGFLPSSEE  399
Fish       387  NEGFGDSNEDDEYELKPYKKKTVGAGRKKM--KKNDSEDEYSPESSD  431
```

B

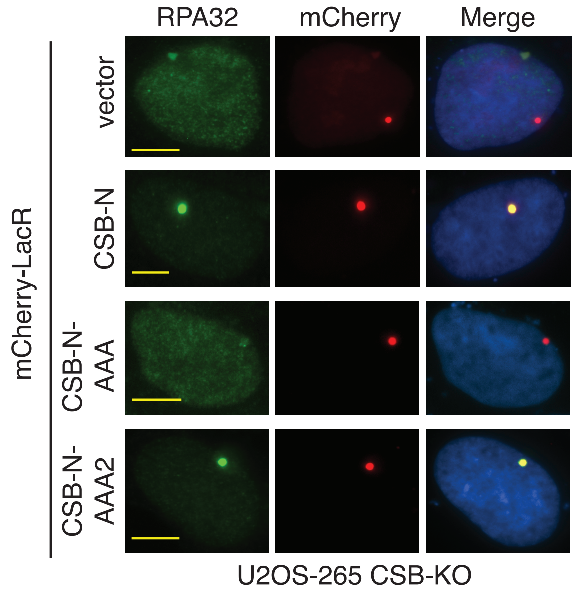

C

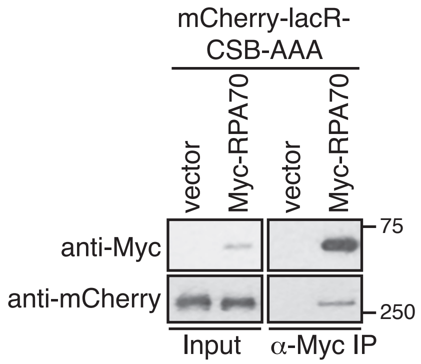

Figure S4. CSB-F uses the highly conserved RQK motif to interact with RPA32C in a manner similar to SMARCAL1 and UNG2. (**A**) Alphafold2 model of RPA32C (yellow) in complex with CSB-F (blue). The RQK motif is highlighted in magenta. Images generated in PyMol v2.5 in this and subsequent panels. (**B**) Alphafold2 model of RPA32C in complex with CSB-F, coloured by pLDDT score. (**C**) Alignment of the Alphafold2 model of RPA32C in complex with CSB-F (coloured as in S3A) with experimental structures of RPA32-UNG2 (pink, PDB 1DPU) and RPA32-SMARCAL1 (light blue, PDB 4MQV). (**D**) Close-up of the binding interface of RPA32C and its interacting partners in (C), with the conserved RQK motif from CSB, UNG2 and SMARCAL1 shown in sticks, and coloured as in (C). (**E**) Comparison of residues in RPA32C interacting with the optimized CSB-RPA32C model with those of UNG2 (PDB 1DPU) and SMARCAL1 (PDB 4MQV). Secondary structure of RPA32C is depicted above its sequence, and interacting residues are shown in Fig. 3A.

Suplementary Figure S4 Batenburg et al.

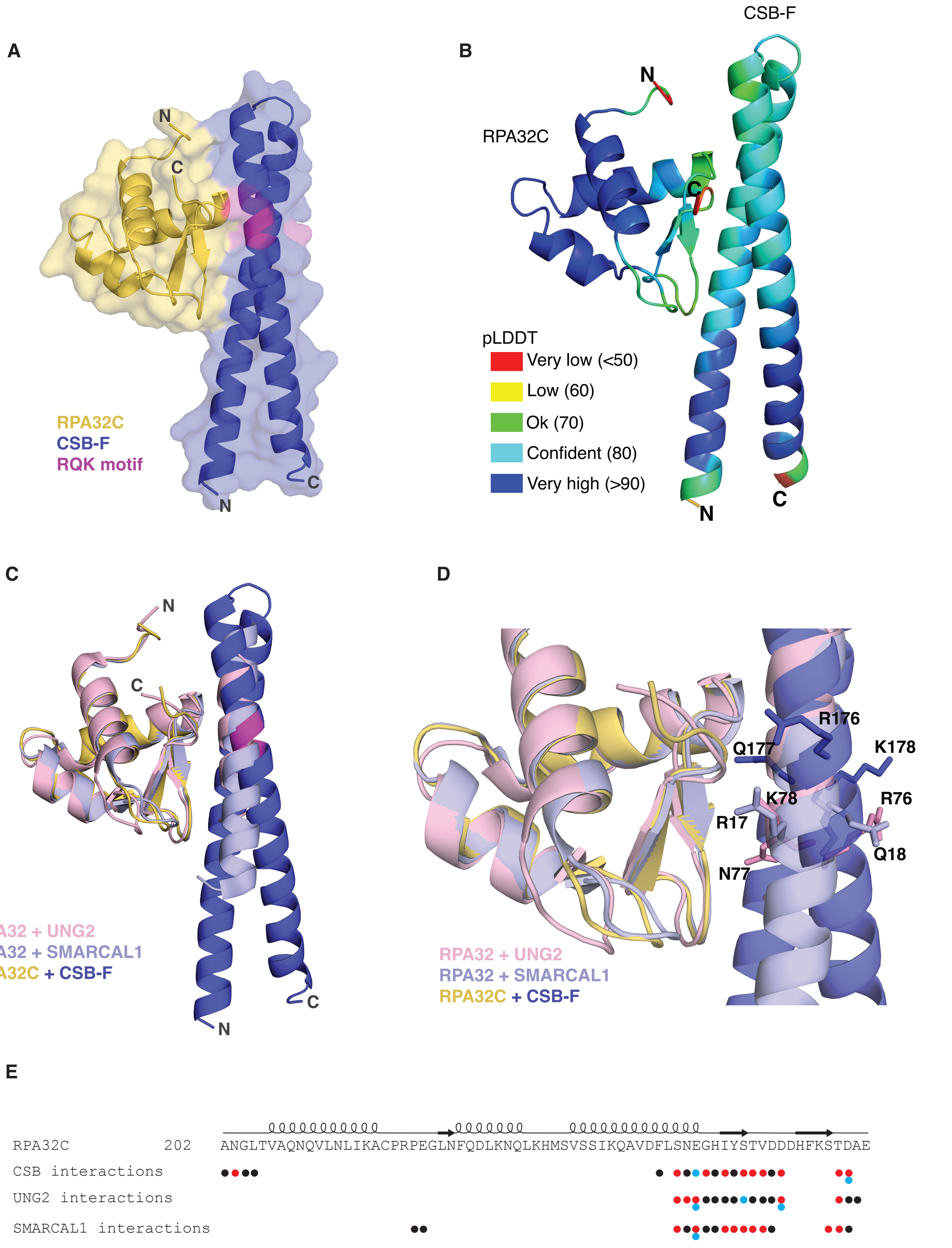

Figure S5. CSB-F exhibits a greater binding affinity to RPA32C than the SMARCAL1 peptide and the $R^{176}Q^{177}K^{178}$ motif contributes to the stability of the CSB-RPA32C interaction. (**A**) Replicate microscale thermophoresis measurements of CSB-F interacting with His-labelled RPA32C. Measurements were taken over 15 seconds, with three independent experiments containing 16 reactions each. Data plotted is the relative fluorescence versus the time of the MST experiment in seconds in this and subsequent panels. (**B**) Replicate microscale thermophoresis measurements of CSB-F-AAA interacting with His-labelled RPA32C. Measurements were taken over 15 seconds, with three independent experiments containing 16 reactions each. (**C**) Replicate microscale thermophoresis measurements of SMARCAL1 interacting with His-labelled RPA32C. Measurements were taken over 15 seconds, with three independent experiments containing 16 reactions each.

A

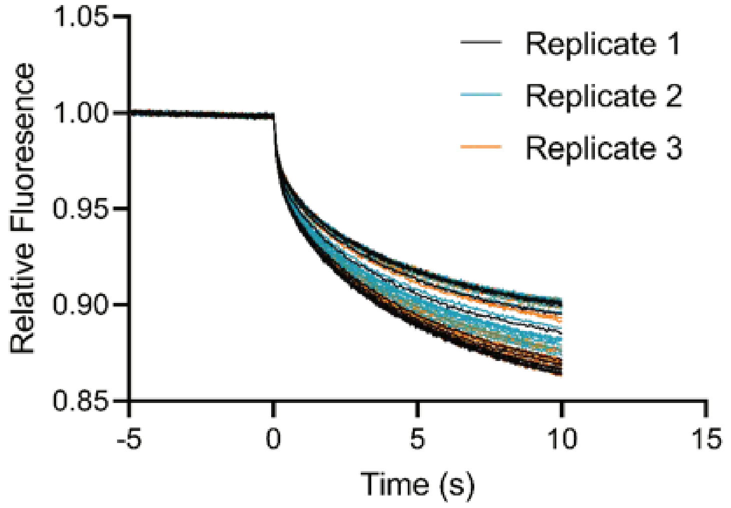
1.05
1.00
0.95
0.90
0.85
Relative Fluoresence
Replicate 1
Replicate 2
Replicate 3
-5
0
5
10
15
Time (s)

B

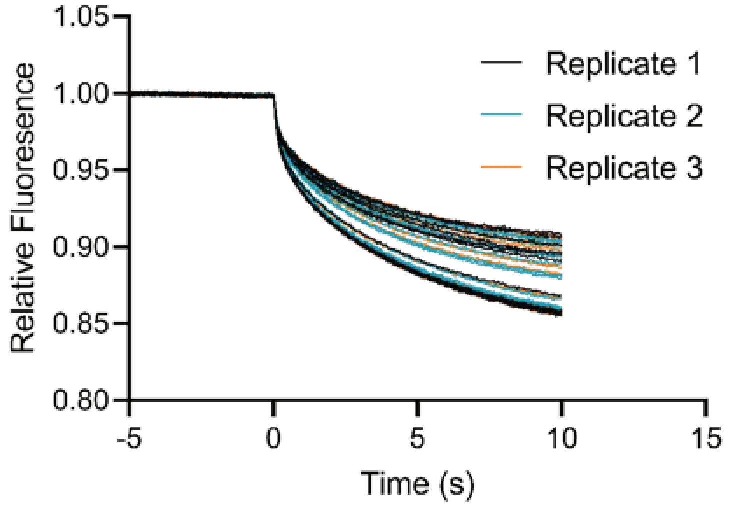
1.05
1.00
0.95
0.90
0.85
0.80
Relative Fluoresence
Replicate 1
Replicate 2
Replicate 3
-5
0
5
10
15
Time (s)

C

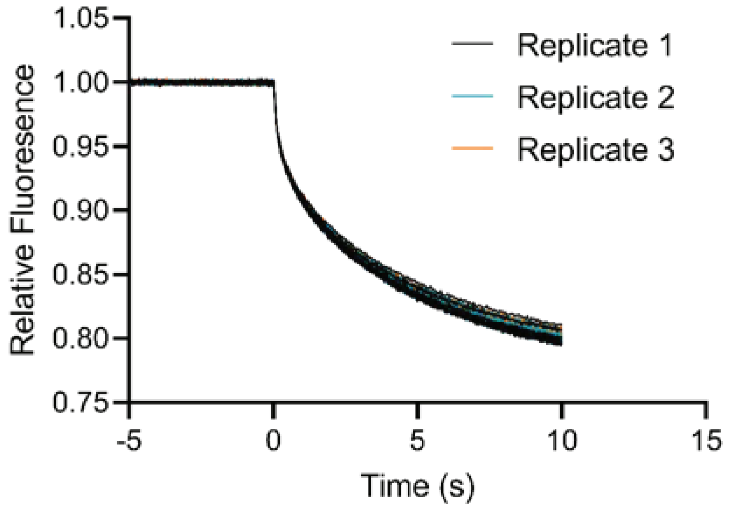
1.05
1.00
0.95
0.90
0.85
0.80
0.75
Relative Fluoresence
Replicate 1
Replicate 2
Replicate 3
-5
0
5
10
15
Time (s)

Figure S6. CSB relies on its $R^{176}Q^{177}K^{178}$ motif for its efficient recruitment to stalled forks as well as for its function in promoting fork slowing and fork degradation in BRCA2-deficient cells. (**A**) Quantification of PLA foci formation between anti-Myc and RPA-pS33 in HU-treated hTERT-RPE CSB-KO cells transfected with the vector alone, Myc-CSB, or Myc-CSB carrying $R^{176}Q^{177}K^{178}$-AAA mutations (AAA). A total of 271-274 cells were scored per condition in a blind manner. Data from single experiments are represented as scatter plot graphs with the mean indicated in this and S6B-S6E panels. The *P*-value was determined using a non-parametric Mann-Whitney rank-sum *t*-test in this and S6B-S6E panels panels. **P<0.01; ***P<0.001. (**B**) Quantification of PLA between anti-Myc and EdU in HU-treated hTERT-RPE CSB-KO cells transfected with the vector alone, Myc-CSB, or Myc-CSB carrying $R^{176}Q^{177}K^{178}$-AAA mutations (AAA). A total of 166-190 cells were scored per condition in a blind manner. *P<0.05; ***P<0.001. (**C**) Quantification of the CldU/IdU ratio from HCT116 CSB-KO cells expressing the vector alone, Myc-CSB, or Myc-CSB carrying $R^{176}Q^{177}K^{178}$-AAA mutations (AAA). A total of 386-460 fibers per condition were analyzed. ***P<0.001. (**D**) Quantification of the CldU/IdU ratio from U2OS CSB-KO cells expressing the vector alone, Myc-CSB, or Myc-CSB carrying the ATPase-dead W851R mutation (W851R). A total of 296-327 fibers per condition were analyzed. ***P<0.001. (**E**) Quantification of the CldU/IdU ratio from siBRCA2-transfected HCT116 CSB-KO cells expressing the vector alone, Myc-CSB, or Myc-CSB carrying $R^{176}Q^{177}K^{178}$-AAA mutations (AAA). A total of 432-449 fibers per condition were analyzed. ***P<0.001.

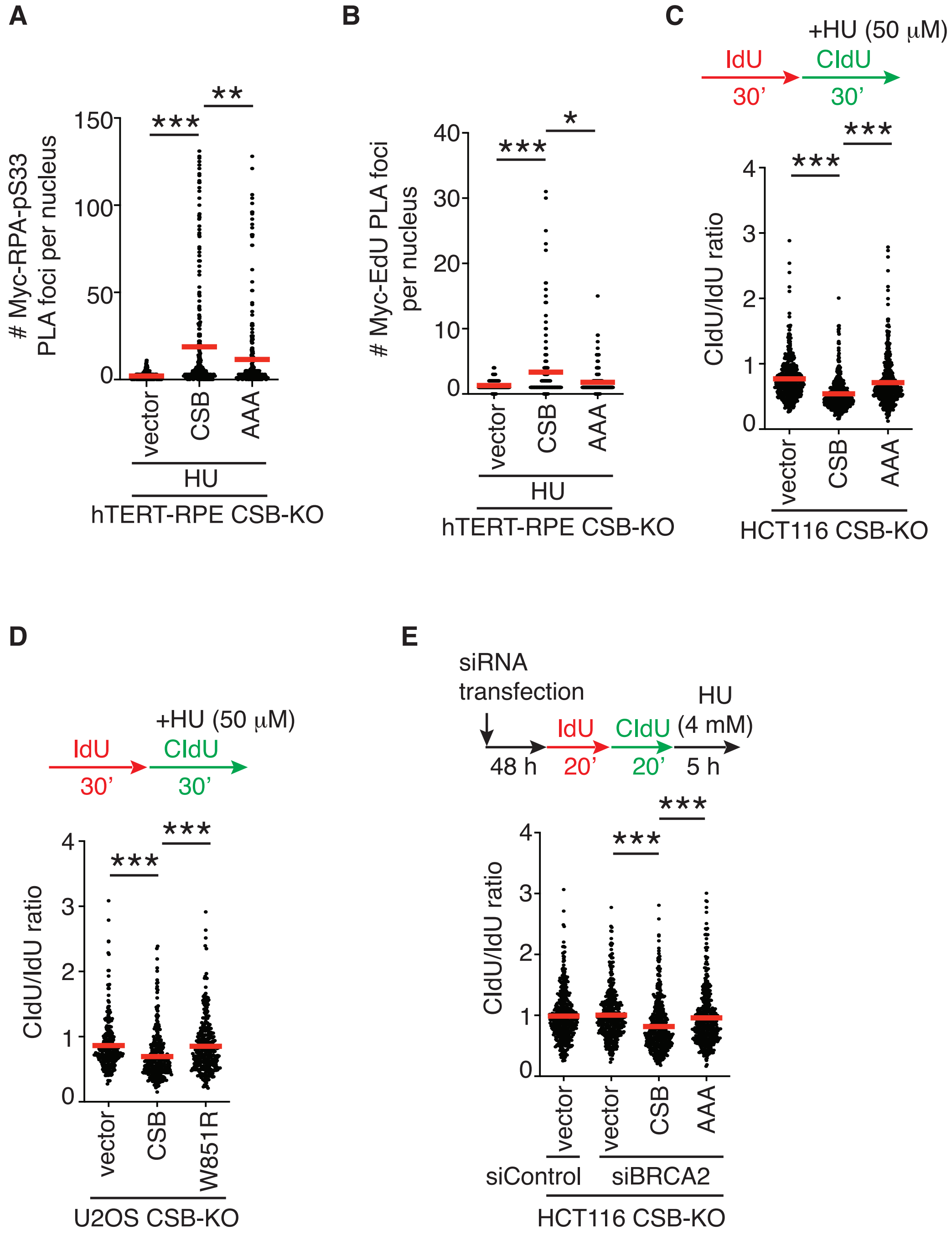
A
Myc-RPA-pS33 PLA foci per nucleus
150
100
50
0
***
**
vector
CSB
AAA
HU
hTERT-RPE CSB-KO
B
Myc-EdU PLA foci per nucleus
40
30
20
10
0
***
*
vector
CSB
AAA
HU
hTERT-RPE CSB-KO
C
+HU (50 µM)
IdU
CldU
30'
30'
CldU/IdU ratio
4
3
2
1
0
***
***
vector
CSB
AAA
HCT116 CSB-KO
D
+HU (50 µM)
IdU
CldU
30'
30'
CldU/IdU ratio
4
3
2
1
0
***
***
vector
CSB
W851R
U2OS CSB-KO
E
siRNA transfection
IdU
CldU
HU (4 mM)
48 h
20'
20'
5 h
CldU/IdU ratio
4
3
2
1
0
***
***
vector
vector
CSB
AAA
siControl
siBRCA2
HCT116 CSB-KO

Figure S7. The $R^{176}Q^{177}K^{178}$ motif of CSB is epistatic to CSB's phosphorylation on T1031 at stalled forks. (**A**) Quantification of PLA foci formation between anti-Myc and EdU in HU-treated U2OS CSB-KO cells transfected with the vector alone, Myc-CSB, Myc-CSB carrying $R^{176}Q^{177}K^{178}$-AAA mutations (AAA), Myc-CSB carrying a T1031A mutation, or Myc-CSCB carrying a combination of $R^{176}Q^{177}K^{178}$-AAA and T1031A mutations. A total of 319-392 cells were scored per condition in a blind manner. Data from single experiments are represented as scatter plot graphs with the mean indicated in this and subsequent panels. The *P*-value was determined using a non-parametric Mann-Whitney rank-sum *t*-test in this and subsequent panels. *$P<0.05$; ***$P<0.001$. (**B**) Quantification of the CldU/IdU ratio from U2OS CSB-KO cells transfected with the vector alone, Myc-CSB, Myc-CSB carrying $R^{176}Q^{177}K^{178}$-AAA mutations (AAA), Myc-CSB carrying a T1031A mutation, or Myc-CSCB carrying a combination of $R^{176}Q^{177}K^{178}$-AAA and T1031A mutations. A total of 324-358 fibers per condition were analyzed. ***$P<0.001$. (**C**) Quantification of the CldU/IdU ratio from BRCA2-depleted U2OS CSB-KO cells transfected with the vector alone, Myc-CSB, Myc-CSB carrying $R^{176}Q^{177}K^{178}$-AAA mutations (AAA), Myc-CSB carrying a T1031A mutation, or Myc-CSCB carrying a combination of $R^{176}Q^{177}K^{178}$-AAA and T1031A mutations. A total of 303-361 fibers per condition were analyzed. ***$P<0.001$.

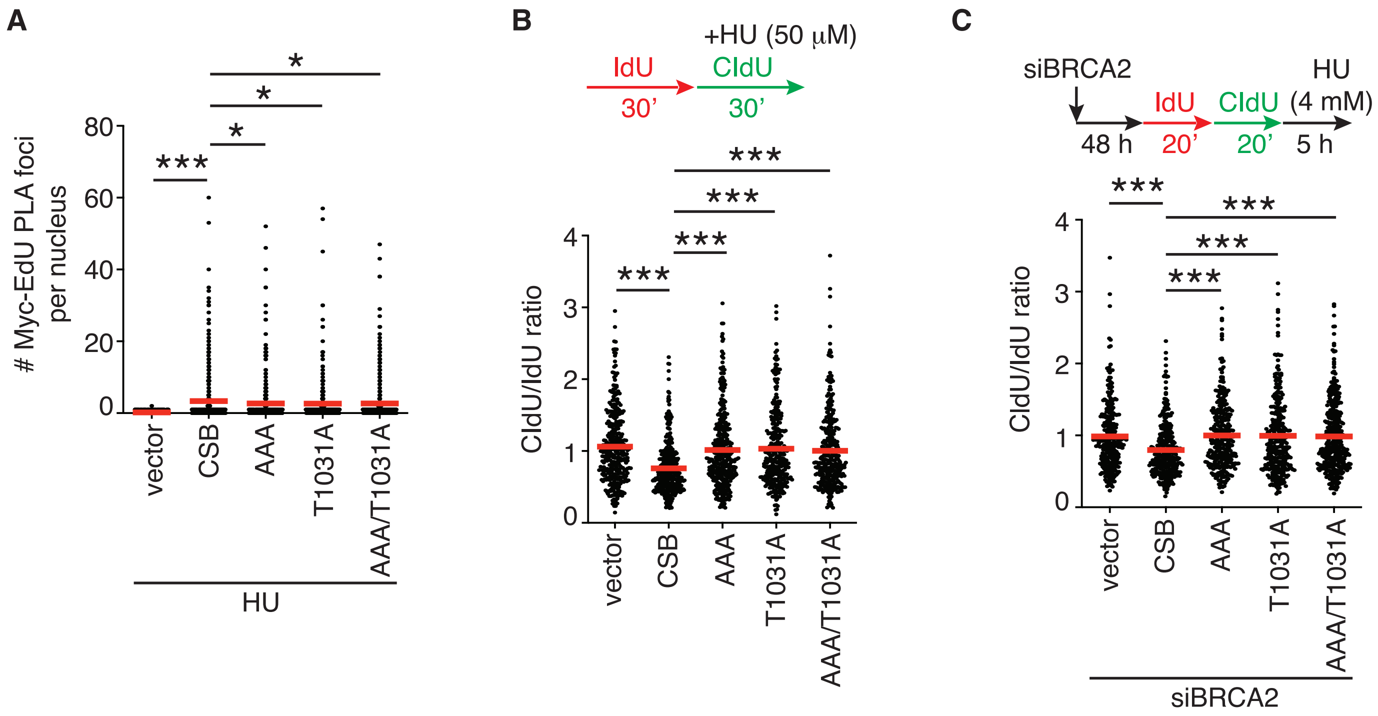
A
Myc-EdU PLA foci per nucleus
0
20
40
60
80
***
*
*
*
vector
CSB
AAA
T1031A
AAA/T1031A
HU
B
+HU (50 μM)
IdU
30'
CldU
30'
CldU/IdU ratio
0
1
2
3
4
***
***
***
***
vector
CSB
AAA
T1031A
AAA/T1031A
C
siBRCA2
48 h
IdU
20'
CldU
20'
HU (4 mM)
5 h
CldU/IdU ratio
0
1
2
3
4
***
***
***
***
vector
CSB
AAA
T1031A
AAA/T1031A
siBRCA2

Figure S8. The $R^{176}Q^{177}K^{178}$ motif of CSB is dispensable for fork restart in both BRCA2-proficient and BRCA2-deficient cells. (**A**) Quantification of the percentage of stalled forks from U2OS CSB-KO cells expressing the vector alone, Myc-CSB, or Myc-CSB carrying $R^{176}Q^{177}K^{178}$-AAA mutations (AAA). A total of 315-363 fibres per condition were scored in a blind manner. SDs from three independent experiments are shown. ***P<0.001. (**B**) Quantification of the percentage of stalled forks from BRCA2-depleted U2OS CSB-KO cells expressing the vector alone, Myc-CSB, or Myc-CSB carrying $R^{176}Q^{177}K^{178}$-AAA mutations (AAA). A total of 306-452 fibres per condition were scored in a blind manner. SDs from three independent experiments are shown. **P<0.01.

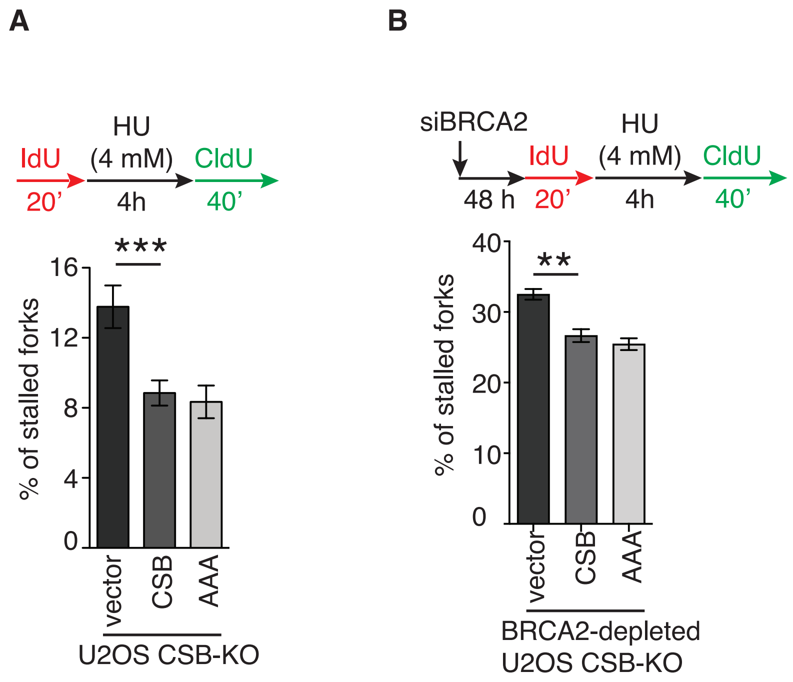
A
HU
IdU
(4 mM)
CldU
20'
4h
40'
***
16
12
8
4
0
% of stalled forks
vector
CSB
AAA
U2OS CSB-KO
B
siBRCA2
HU
IdU
(4 mM)
CldU
48 h
20'
4h
40'
40
30
20
10
0
**
% of stalled forks
vector
CSB
AAA
BRCA2-depleted
U2OS CSB-KO
